# Genetic variants of *MUC4* are associated with susceptibility to and mortality of colorectal cancer and exhibit synergistic effects with LDL-C levels

**DOI:** 10.1371/journal.pone.0287768

**Published:** 2023-06-29

**Authors:** Min Jung Kwon, Jeong Yong Lee, Eo Jin Kim, Eun Ju Ko, Chang Soo Ryu, Hye Jung Cho, Hak Hoon Jun, Jong Woo Kim, Nam Keun Kim

**Affiliations:** 1 Department of Biomedical Science, College of Life Science, CHA University, Seongnam, South Korea; 2 Division of Hematology/Oncology, Department of Internal Medicine, Kangbuk Samsung Hospital, Sungkyunkwan University School of Medicine, Seoul, South Korea; 3 Department of Surgery, CHA Bundang Medical Center, CHA University, Seongnam, South Korea; German Cancer Research Center (DKFZ), GERMANY

## Abstract

As a disease with high mortality and prevalence rates worldwide, colorectal cancer (CRC) has been thoroughly investigated. Mucins are involved in the induction of CRC and the regulation of intestinal homeostasis but a member of the mucin gene family *MUC4* has a controversial role in CRC. *MUC4* has been associated with either decreased susceptibility to or a worse prognosis of CRC. In our study, the multifunctional aspects of *MUC4* were elucidated by genetic polymorphism analysis in a case-control study of 420 controls and 464 CRC patients. *MUC4* rs1104760 A>G polymorphism had a protective effect on CRC risk (AG, AOR = 0.537; GG, AOR = 0.297; dominant model, AOR = 0.493; recessive model, AOR = 0.382) and *MUC4* rs2688513 A>G was associated with an increased mortality rate of CRC (5 years, GG, adjusted HR = 6.496; recessive model, adjusted HR = 5.848). In addition, *MUC4* rs1104760 A>G showed a high probability of being a potential biomarker for CRC patients with low-density lipoprotein cholesterol (LDL-C) in the risk range while showing a significant synergistic effect with the LDL-C level. This is the first study to indicate a significant association between *MUC4* genetic polymorphisms and CRC prevalence, suggesting a functional genetic variant with the LDL-C level, for CRC prevention.

## Introduction

Colorectal cancer (CRC) is the third most common cancer and the fourth leading cause of cancer-related death worldwide [1]. Because of its high prevalence and mortality rates, many researchers have studied the molecular mechanisms of CRC but the frequency of occurrence and death rate of CRC is still high, and its clinical treatment via surgery remains stagnant [2]. Therefore, new treatment concepts including microsatellite instability (MSI) and KRAS or BRAF mutations have become an area of focus for identifying the genetic causes of CRC and improving personalized medicine for CRC patients [3, 4]. CRC is a highly prevalent malignancy with multifactorial etiology, which includes metabolic alterations as contributors to disease development. However, few studies have shown an association between genetic variants and metabolic factors due to the complexity of the correlations among them. Therefore, we examined the correlations between genetic variants of a well-known CRC-related gene, *MUC4*, and CRC prevalence with regard to metabolic factors.

Mucins are a family of molecules responsible for the protection, repair, and survival of epithelial tissue in the intestines [5]. They maintain the homeostasis and physiological environment of the gut by preventing the invasion of pathogens. The members of the mucin family genes have indicated an abnormal expression in CRC. MUC1 [6] and MUC13 [7] act as oncogenes, and MUC2 [8] and MUC6 [9] act as tumor suppressors. Interestingly, *MUC4* also shows aberrant expression in many epithelial tissues including CRC, but the role of *MUC4* in CRC is controversial because studies of *MUC4* expression in CRC have shown conflicting results. While some studies have suggested that loss or reduction of *MUC4* expression occurs in CRC [10], other studies have suggested that the majority (approximately 75%) of CRC tumors have a decreased level or loss of *MUC4* expression, and a subset (approximately 25%) of CRC tumors have high *MUC4* expression [11, 12].

Although many previous studies have indicated aberrant *MUC4* expression in CRC carcinogenesis, only one study showed an association between genetic polymorphisms of *MUC4* and the prognosis of CRC [13–15]. Therefore, we analyzed single nucleotide polymorphisms (SNPs) of *MUC4* in CRC patients in the Korean population. We selected *MUC4* rs882605, rs1104760, and rs26885813 which were suggested to have a significant function on *MUC4* expression in our previous paper [16], and selected rs2246901 that was associated with epithelial tumor [17]. We analyzed genetic associations between *MUC4* SNPs and CRC prevalence as well as metabolic factors to determine potential biomarkers for CRC development. The present paper is the first study to suggest that genetic variants of *MUC4* play important roles in the prevalence and prognosis of CRC while suggesting a significant synergistic effect between the low-density lipid cholesterol (LDL-C) level and *MUC4* polymorphism against CRC prevalence. Considering that novel medical treatments are needed for CRC therapy, this study will provide a new perspective to initiate personalized medicine for the diagnosis and treatment of CRC.

## Materials and methods

### Study population

This case-control study included 884 individuals enrolled between 1996 and 2009 and was reviewed and approved by the Institutional Review Board of CHA Bundang Medical Center (IRB No. 2009–08–077) on October 20 in 2009. Samples data were accessed from October 2021 to April 2022 for the purpose of our study. The 464 CRC patients were diagnosed at the CHA Bundang Medical Center (Seongnam, South Korea), had histologically proven adenocarcinoma, and had undergone surgical resection with curative intent. The patients included 260 colon cancer patients, 192 rectal cancer patients, and 12 consecutive patients with unclassified CRC who had undergone primary surgery. Tumor classification was conducted according to the tumor, node, and metastasis classification staging system of the 7th American Joint Committee on Cancer staging manual. Hypertension and diabetes mellitus were classified using the same criteria used in our previous study [18]. The controls included 424 individuals who were randomly selected from a health screening program, and participants with a history of thrombotic diseases or cancers were excluded. All participants were Korean and provided written informed consent. Our study followed the recommendations of the Declaration of Helsinki.

### Genotyping

DNA was extracted from white blood cells using a G-DEX II Genomic DNA Extraction kit (iNtRON Biotechnology, South Korea). The SNPs of *MUC4* were determined based on our previous studies and articles in the literature. TaqMan allele discrimination analysis was used to determine the genotypes, and the analysis protocol was the same as that used in our previous study [19]. We randomly chose about 10% of the samples to confirm the results and performed sequencing. The concordance between the experimental results and randomly repeated samples was 100%.

### Statistical analysis

For comparisons of baseline characteristics between the CRC and control groups, chi-square tests and Student’s t-tests were used to assess categorical and continuous data, respectively. All genotype frequencies of polymorphisms were in Hardy-Weinberg equilibrium (HWE) for both controls and patients, and these polymorphisms were analyzed in reference to the wild-type genotype. Multivariate logistic regression was applied to estimate the association of *MUC4* polymorphisms with CRC occurrence using adjusted odds ratios (AORs) and 95% confidence intervals (CIs) that were adjusted for age, gender, hypertension, diabetes mellitus, body mass index (BMI), and HDL-C levels. These adjustment variables were selected because they are risk factors for metabolic syndrome that affects CRC. Additionally, ROC curve analysis was conducted to assess the relationship between genetic polymorphisms and disease status, and subgroup analyses were performed for a range of environmental factors. An area under the curve (AUC) of approximately 1.00 indicated that a variable was a precise biomarker for CRC, while an AUC of 0.50 indicated that the variable was not an accurate biomarker. In general, an AUC greater than 0.60 indicated that a variant was a significant biomarker for the disease. The associations between clinical characteristics and genetic polymorphisms were assessed using an analysis of variance.

The effects of correlations between environmental factors and genetic variants on CRC were analyzed via interaction analyses and stratified analyses. Spearman correlation analysis was also conducted to show the effects of correlations between lipid-related factors, such as the correlation between HDL-C and LDL-C, after adjustment for age, sex, and BMI. Survival analysis was implemented using the Cox proportional hazards model. Survival was measured using the same method used in our previous study [20]. The results were adjusted for age, sex, presence of hypertension, presence of diabetes mellitus, tumor size, tumor differentiation, chemotherapy, smoking, and alcohol use. We excluded 100 CRC patients who had an insufficient medical history. Overall survival was defined as the time from surgery to death or the final follow-up, and relapse-free survival was defined as the time from surgery to cancer relapse or the final follow-up. Participants were followed for a median of 34 months (range, 4–173 months). Hazard ratios (HRs) are presented with 95% CIs. All analyses were conducted using Medcalc version 12.7.1.0 (Medcalc Software, Mariakerke, Belgium) and GraphPad Prism 4.0 (GraphPad Software Inc., San Diego, CA, USA). *P*-values < 0.05 were regarded as significant, and the false discovery rate (FDR) method was used to estimate the overall experimental error rate. The FDR method provides a measure of the expected proportion of false positives among data; therefore, FDR-*P* < 0.05 means more powerful statistical significance.

## Results

### Comparison of baseline characteristics between CRC patients and controls

The baseline characteristics were evaluated in controls and colorectal, colon, and rectal cancer patients (Table 1). Before analysis, the chi-square test and t-test were conducted to adjust the age and sex of the control group according to the age and sex of the case group. We randomly matched controls to cases and confirmed that age and sex were matched between patients and controls by showing a *P*-value > 0.05. Regarding lipid level-related factors, a difference between controls and each of the three types of cancer patients was statistically significant, except for the folate and triglyceride levels of rectal cancer patients. The levels of lipid-related factors were significantly lower in all types of cancer patients than those in healthy subjects.

**Table 1:** Baseline characteristics between patients and control subjects.

Characteristic	Control (n = 420)	CRC (n = 464)	*P*	Colon (n = 260)	*P*	Rectum (n = 192)	*P*
Age (years, mean ± SD)	60.85±11.70	61.62±12.53	0.349	61.29±13.24	0.425	61.59±11.50	0.465
Male (%)	168 (40.0)	212 (45.7)	0.101	114 (17.6)	0.363	91 (9.2)	0.103
HTN (%)	163 (38.8)	293 (63.1)	**< 0.0001**	161 (24.3)	**< 0.0001**	124 (12.7)	**< 0.0001**
DM (%)	50 (11.9)	150 (32.3)	**< 0.0001**	88 (12.4)	**< 0.0001**	62 (6.5)	**< 0.0001**
Smoking (%)	137 (32.6)	89 (19.2)	**0.0001**	52 (7.4)	**0.002**	35 (3.9)	**0.002**
BMI (kg/m^2^, mean ± SD)	24.24±3.35	23.22±3.22	**< 0.0001**	23.10±3.33	**0.002**	23.32±3.06	**0.014**
Hcy (μmol/L, mean ± SD)	9.74±4.06	10.53±7.74	0.368	10.36±8.24	0.866	10.67±7.08	0.122
Folate (nmol/L, mean ± SD)	8.93±7.79	7.83±6.82	**< 0.0001**	7.96±6.84	**0.001**	7.64±6.86	0.070
TG (mg/dL, mean ± SD)	145.92±89.96	131.47±87.37	**0.022**	130.90±87.29	**0.044**	132.61±89.15	0.114
T.chol (mg/dL, mean ± SD)	191.78±36.65	176.21±40.38	**< 0.0001**	174.06±37.32	**< 0.0001**	176.74±44.02	**0.001**
HDL-C (mg/dL, mean ± SD)	46.02±13.95	42.27±12.86	**0.003**	42.34±12.78	**0.007**	42.19±13.01	**0.007**
LDL-C (mg/dL, mean ± SD)	116.28±39.84	100.52±26.59	**0.0005**	99.74±26.07	**0.002**	100.50±27.45	**0.014**
Tumor size (%)							
<5cm		188 (40.5)		90 (34.6)		95 (49.5)	
≥5cm		264 (56.9)		165 (63.5)		97 (50.5)	
TNM stage (%)							
Ⅰ		48 (10.3)		22 (8.5)		26 (10.0)	
Ⅱ		183 (39.4)		113 (43.5)		66 (25.4)	
Ⅲ		182 (39.2)		99 (38.1)		82 (31.5)	
Ⅳ		46 (9.9)		25 (9.6)		18 (6.9)	
MSI (%)							
MSI-high (%)		42 (9.1)		36 (13.8)		6 (2.3)	
MSI-low (%)		16 (3.4)		11 (4.2)		5 (1.9)	

CRC, colorectal cancer; SD, standard deviation; HTN, hypertension; DM, diabetes mellitus; BMI, body mass index; Hcy, plasma homocysteine; TG, triglyceride; T.chol, total cholesterol; HDL-C, high-density lipoprotein cholesterol; LDL-C, low-density lipoprotein cholesterol; TNM stage, tumor node metastasis; MSI, microsatellite instability.

*P*-values were calculated using chi-squared tests for categorical data and two-sided t-tests for continuous data.

### *MUC4* rs1104760 A>G and rs2688513 A>G polymorphisms are associated with decreased susceptibility to CRC

The effects of four *MUC4* polymorphisms (rs882605 G>T, rs1104760 A>G, rs2688513 A>G, and rs2246901 A>C) on CRC risk were evaluated and age, sex, hypertension, diabetes mellitus, body mass index (BMI), and high-density lipoprotein cholesterol (HDL-C) levels were adjusted (Table 2). *MUC4* rs1104760 G allele had a protective effect against CRC occurrence compared to the A allele (AG, AOR = 0.537, *P* = 0.010, FDR*-P* = 0.040; GG, AOR = 0.297, *P* = 0.008, FDR*-P* = 0.032; AA vs AG+GG, AOR = 0.493, *P* = 0.002, FDR*-P* = 0.008; AA+AG vs GG, AOR = 0.382, *P* = 0.027, FDR*-P* = 0.108). When cases were classified by tumor location as colon cancer or rectal cancer, the majority of the findings remained statistically significant (in colon cancer: AG, AOR = 0.433, 95% CI = 0.251–0.747, *P* = 0.003, FDR*-P* = 0.012; GG, AOR = 0.363, 95% CI = 0.137–0.963, *P* = 0.042, FDR*-P* = 0.168; AA vs AG+GG, AOR = 0.424, 95% CI = 0.254–0.708, *P* = 0.001, FDR*-P* = 0.004, and in rectal cancer: GG, AOR = 0.382, 95% CI = 0.163–0.898 *P* = 0.015; AA vs AG+GG, AOR = 0.180, 95% CI = 0.045–0.717, *P* = 0.040; AA+AG vs GG, AOR = 0.562, 95% CI = 0.325–0.973, *P* = 0.022). Notably, the association between colon cancer prevalence and the heterozygous genotype and the dominant model, respectively, remained significant after the FDR test. *MUC4* rs2688513 AG genotype was significantly less frequent in the colon cancer group, although its significance was not maintained in the FDR*-P* test. However, the frequencies of *MUC4* rs882605 G>T and rs2246901 A>C polymorphisms did not show a remarkable association with CRC susceptibility.

**Table 2:** Comparison of genotype frequencies and AOR values of polymorphisms between the CRC and control subjects.

Genotypes	Controls (n = 420)	CRC (n = 464)	AOR (95% CI)	*P*	FDR*-P*	Colon (n = 260)	AOR (95% CI)	*P*	FDR*-P*	Rectum (n = 192)	AOR (95% CI)	*P*	FDR*-P*
*MUC4* rs882605 G>T													
GG	247 (53.7)	283 (48.8)	1.000 (reference)			158 (52.7)	1.000 (reference)			119 (50.0)	1.000 (reference)		
GT	153 (33.3)	163 (28.1)	1.064 (0.658–1.722)	0.801	0.874	89 (29.7)	0.854 (0.493–1.479)	0.573	0.573	69 (29.0)	1.250 (0.705–2.215)	0.445	0.593
TT	20 (4.3)	18 (3.1)	0.669 (0.187–2.396)	0.537	0.960	13 (4.3)	0.772 (0.198–3.014)	0.710	0.759	4 (1.7)	0.374 (0.060–2.314)	0.290	0.387
Dominant			1.031 (0.648–1.642)	0.897	0.911		0.866 (0.512–1.466)	0.592	0.592		1.161 (0.665–2.028)	0.600	0.867
Recessive			0.677 (0.193–2.374)	0.543	0.932		0.907 (0.239–3.444)	0.886	0.886		0.347 (0.057–2.100)	0.249	0.332
HWE-*P*	0.549	0.358											
*MUC4* rs1104760 A>G													
AA	202 (43.9)	260 (44.8)	1.000 (reference)			150 (50.0)	1.000 (reference)			108 (45.4)	1.000 (reference)		
AG	175 (38.0)	180 (31.0)	0.537 (0.334–0.863)	**0.010**	**0.040**	93 (31.0)	0.433 (0.251–0.747)	**0.003**	**0.012**	78 (32.8)	0.653 (0.371–1.150)	0.140	0.560
GG	43 (9.3)	24 (4.1)	0.297 (0.121–0.730)	**0.008**	**0.032**	17 (5.7)	0.363 (0.137–0.963)	**0.042**	0.168	6 (2.5)	0.180 (0.045–0.717)	**0.015**	0.060
Dominant			0.493 (0.313–0.775)	**0.002**	**0.008**		0.424 (0.254–0.708)	**0.001**	**0.004**		0.562 (0.325–0.973)	**0.040**	0.160
Recessive			0.382 (0.163–0.898)	**0.027**	0.108		0.512 (0.202–1.295)	0.157	0.628		0.209 (0.055–0.798)	**0.022**	0.088
HWE-*P*	0.576	0.315											
*MUC4* rs2688513 A>G													
AA	249 (54.1)	281 (48.4)	1.000 (reference)			163 (54.3)	1.000 (reference)			113 (47.5)	1.000 (reference)		
AG	154 (33.5)	164 (28.3)	0.778 (0.486–1.245)	0.295	0.590	85 (28.3)	0.562 (0.326–0.971)	**0.039**	0.078	73 (30.7)	0.990 (0.564–1.736)	0.971	0.971
GG	17 (3.7)	19 (3.3)	1.200 (0.307–4.699)	0.793	0.960	12 (4.0)	1.257 (0.291–5.428)	0.759	0.759	6 (2.5)	0.940 (0.177–4.985)	0.942	0.942
Dominant			0.809 (0.512–1.278)	0.363	0.726		0.620 (0.367–1.046)	0.073	0.146		0.990 (0.572–1.714)	0.971	0.971
Recessive			1.300 (0.343–4.925)	0.699	0.932		1.546 (0.372–6.423)	0.549	0.732		0.961 (0.193–4.782)	0.961	0.961
HWE *P*	0.257	0.416											
*MUC4* rs2246901 A>C													
AA	255 (55.4)	273 (47.1)	1.000 (reference)			157 (52.3)	1.000 (reference)			112 (47.1)	1.000 (reference)		
AC	137 (29.8)	166 (28.6)	0.962 (0.597–1.550)	0.874	0.874	83 (27.7)	0.665 (0.383–1.157)	0.149	0.199	76 (31.9)	1.274 (0.725–2.240)	0.400	0.593
CC	28 (6.1)	25 (4.3)	1.030 (0.332–3.198)	0.960	0.960	20 (6.7)	1.518 (0.473–4.872)	0.483	0.759	4 (1.7)	0.242 (0.039–1.497)	0.127	0.254
Dominant			0.974 (0.617–1.538)	0.911	0.911		0.770 (0.459–1.292)	0.322	0.429		1.136 (0.655–1.967)	0.650	0.867
Recessive			0.965 (0.321–2.902)	0.950	0.950		1.626 (0.522–5.069)	0.402	0.732		0.202 (0.035–1.180)	0.076	0.152
HWE-*P*	0.108	0.971											

CRC, colorectal cancer; AOR, adjusted odds ratio; 95% CI, 95% confidence interval; HWE, Hardy-Weinberg equilibrium; N/A, not applicable.

AOR adjusted by age, sex, hypertension, diabetes mellitus, body mass index, high-density lipoprotein cholesterol.

Moreover, because MSI is closely related to CRC and MSI-high status is an emerging predictive and prognostic biomarker for the immunotherapy response in cancer [21], we measured the associations of *MUC4* polymorphisms with MSI status (Table 3). The *MUC4* rs1104760 AG and GG genotypes and the dominant model had a protective effect in MSI patients and the dominant model maintained a significant *P*-value after adjusting for the FDR. Interestingly, in MSI-high-status patients, both the heterozygous genotype and dominant model of *MUC4* rs882605, rs1104760, and rs2688513 indicated a significant association with CRC occurrence while all *MUC4* polymorphisms did not show significance in MSI-low status.

**Table 3:** Comparison of genotype frequencies and AOR values of polymorphisms between the MSI status and control subjects.

Genotypes	Controls (n = 420)	MSI (n = 58)	AOR (95% CI)	*P*	FDR*-P*	MSI-high (n = 42)	AOR (95% CI)	*P*	FDR*-P*	MSI-low (n = 16)	AOR (95% CI)	*P*	FDR*-P*
*MUC4* rs882605G>T													
GG	247 (53.7)	39 (67.2)	1.000 (reference)			29 (69.0)	1.000 (reference)			10 (62.5)	1.000 (reference)		
GT	153 (33.3)	15 (25.9)	0.524 (0.216–1.272)	0.153	0.204	9 (21.4)	0.292 (0.088–0.972)	**0.045**	0.060	6 (37.5)	1.012 (0.308–3.328)	0.985	0.985
TT	20 (4.3)	4 (6.9)	0.217 (0.022–2.162)	0.193	0.257	4 (9.5)	0.324 (0.032–3.284)	0.340	0.453	0 (0.0)	N/A		
Dominant			0.487 (0.206–1.148)	0.100	0.115		0.297 (0.097–0.909)	**0.033**	0.051		0.872 (0.269–2.830)	0.820	0.946
Recessive			0.375 (0.047–3.000)	0.356	0.475		0.496 (0.055–4.469)	0.532	0.709		N/A		
*MUC4* rs1104760A>G													
AA	202 (43.9)	35 (60.3)	1.000 (reference)			26 (61.9)	1.000 (reference)			9 (56.3)	1.000 (reference)		
AG	175 (38.0)	18 (31.0)	0.392 (0.171–0.900)	**0.027**	0.108	11 (26.2)	0.257 (0.088–0.748)	**0.013**	0.052	7 (43.8)	0.721 (0.226–2.300)	0.581	0.985
GG	43 (9.3)	5 (8.6)	0.150 (0.025–0.907)	**0.039**	0.156	5 (11.9)	0.217 (0.034–1.397)	0.108	0.432	0 (0.0)	N/A		
Dominant			0.351 (0.158–0.779)	**0.010**	**0.040**		0.260 (0.096–0.703)	**0.008**	**0.032**		0.565 (0.180–1.777)	0.329	0.946
Recessive			0.289 (0.060–1.383)	0.120	0.422		0.436 (0.082–2.307)	0.329	0.709		N/A		
*MUC4* rs2688513A>G													
AA	249 (54.1)	36 (62.1)	1.000 (reference)			27 (64.3)	1.000 (reference)			9 (56.3)	1.000 (reference)		
AG	154 (33.5)	18 (31.0)	0.516 (0.226–1.179)	0.116	0.204	11 (26.2)	0.335 (0.116–0.963)	**0.042**	0.060	7 (43.8)	1.048 (0.332–3.315)	0.936	0.985
GG	17 (3.7)	4 (6.9)	0.293 (0.025–3.443)	0.329	0.329	4 (9.5)	0.491 (0.041–5.930)	0.576	0.576	0 (0.0)	N/A		
Dominant			0.498 (0.220–1.127)	0.095	0.115		0.336 (0.120–0.941)	**0.038**	0.051		0.961 (0.307–3.012)	0.946	0.946
Recessive			0.528 (0.057–4.917)	0.575	0.575		0.717 (0.066–7.840)	0.785	0.785		N/A		
*MUC4* rs2246901A>C													
AA	255 (55.4)	37 (63.8)	1.000 (reference)			27 (64.3)	1.000 (reference)			10 (62.5)	1.000 (reference)		
AC	137 (29.8)	17 (29.3)	0.587 (0.253–1.362)	0.215	0.215	11 (26.2)	0.427 (0.149–1.225)	0.114	0.114	6 (37.5)	0.935 (0.278–3.152)	0.914	0.985
CC	28 (6.1)	4 (6.9)	0.171 (0.019–1.519)	0.113	0.226	4 (9.5)	0.299 (0.033–2.749)	0.286	0.453	0 (0.0)	N/A		
Dominant			0.518 (0.228–1.175)	0.115	0.115		0.395 (0.143–1.089)	0.073	0.073		0.743 (0.226–2.450)	0.626	0.946
Recessive			0.280 (0.038–2.056)	0.211	0.422		0.386 (0.047–3.199)	0.378	0.709		N/A		

MSS, microsatellite stable; MSI, microsatellite instability; AOR, adjusted odds ratio; 95% CI, 95% confidence interval; N/A, not applicable.

AOR adjusted by age, sex, hypertension, diabetes mellitus, body mass index, high-density lipoprotein cholesterol.

### Correlation between the *MUC4* rs1104760 A>G and the HDL-C and LDL-C concentrations regarding susceptibility to CRC

**Fig 1:**
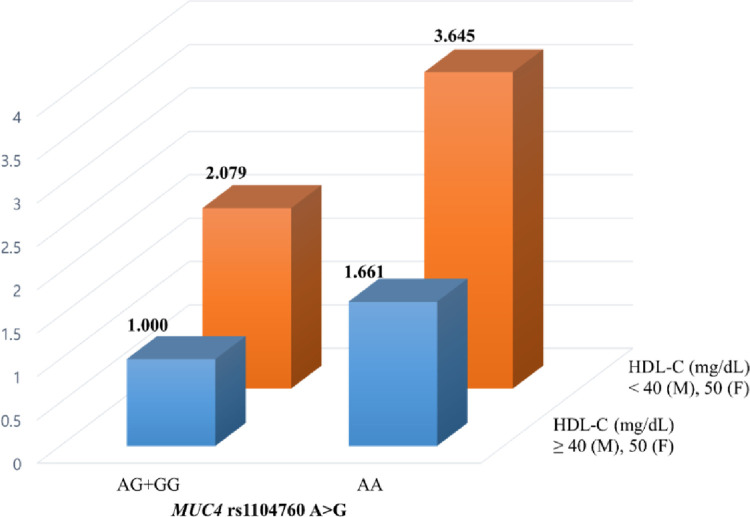
Effects of *MUC4* rs1104760 A>G variant on colorectal cancer (CRC) risk as modulated by high-density lipoprotein cholesterol (HDL-C) levels, hypertension (HTN), and diabetes mellitus (DM). Each synergistic effect is presented as an adjusted odds ratio. The CRC risk was significantly increased when *MUC4* rs1104760 AA variant was present compared with the AG+GG variant. In particular, the combination of the *MUC4* rs1104760 AA variant and an HDL-C level in the risk range resulted in an approximately 4-fold increase in CRC prevalence compared with the presence of the AG+GG variant and an HDL-C level in the normal range.

**Fig 2:**
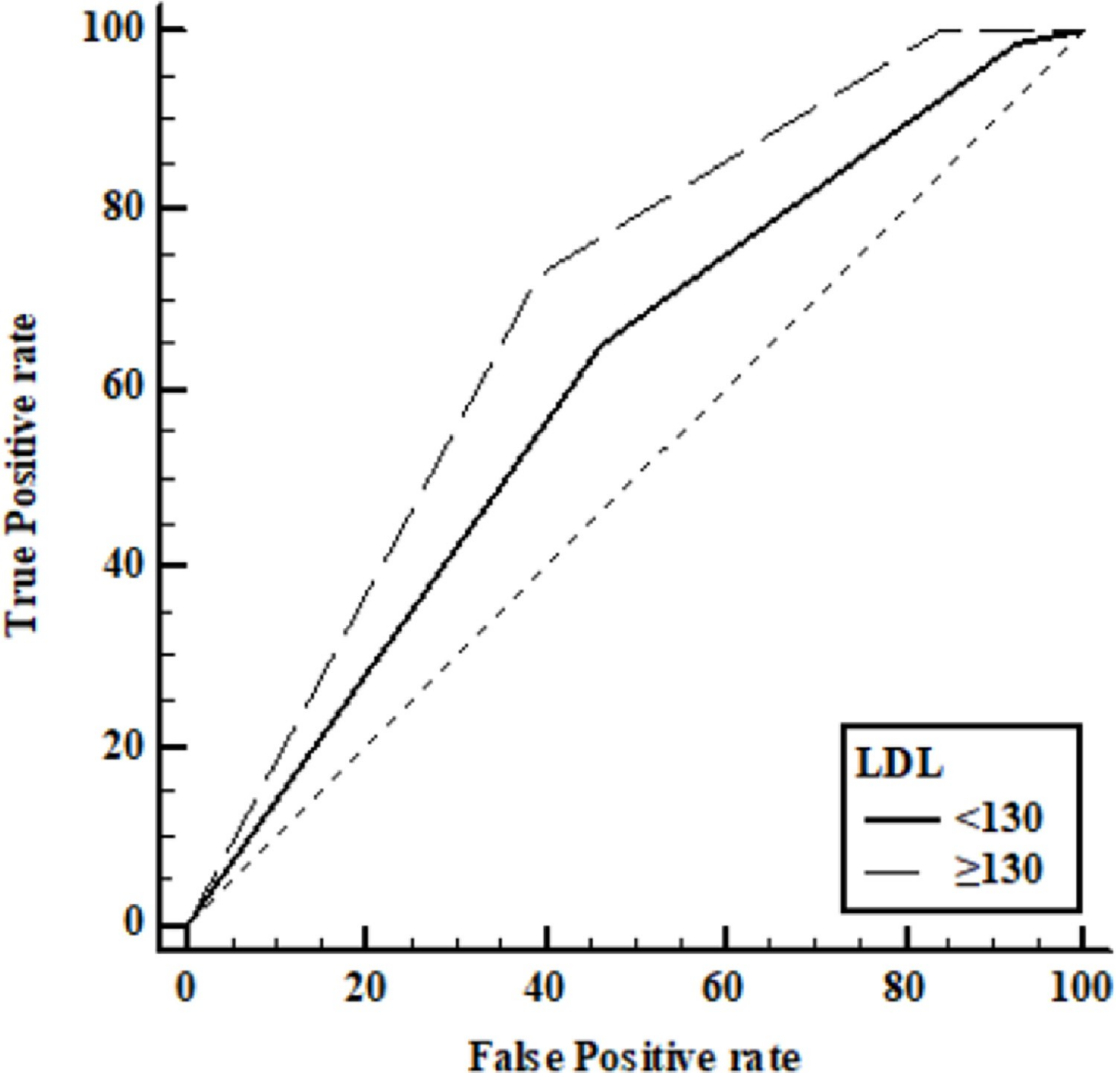
Low-density lipoprotein cholesterol (LDL-C) level-dependent receiver operating characteristic (ROC) curve analysis of *MUC4* rs1104760 A>G and colorectal cancer risk. Each independent ROC curve was computed based on the LDL-C range; pairwise comparisons of the area under the curve (AUC) were performed. The ROC curve including *MUC4* rs1104760 A>G and LDL-C levels in the risk range showed a better predictive value (AUC = 0.689) than that including LDL-C levels in the normal range (AUC = 0.603).

As CRC is a complex disease affected by various environmental factors, the synergistic effects of clinical parameters and *MUC4* polymorphisms for CRC risk were assessed by performing stratified analysis and interaction analysis (Table 4, S1 Table). Interestingly, the *MUC4* rs1104760 AA variant exhibited a stronger synergistic effect with LDL-C levels than did the GG+AG variant. When the *MUC4* rs1104760 AA variant was combined with LDL-C levels in the risk range, CRC occurrence was increased approximately 5-fold compared with that in patients with this variant and LDL-C levels in the normal range (Table 4). In the interaction analysis, the HDL-C levels, which are closely related to LDL-C levels, had synergistic effects with the *MUC4* rs1104760 AA genotype, showing significantly increased CRC risk when combined with the AA genotype (Fig 1). In addition, the LDL-C and HDL-C levels showed a positive correlation in a partial Spearman correlation analysis (ρ = 0.244) although it was a weak correlation. Furthermore, in a receiver operating characteristic (ROC) curve analysis, *MUC4* rs1104760 A>G was indicated as a possible biomarker for CRC patients with high LDL-C levels (area under the curve (AUC) = 0.689) compared to those with normal LDL-C levels (AUC = 0.603) (Fig 2).

**Table 4:** Stratified effects of *MUC4* polymorphisms on CRC susceptibility.

Characteristics	rs882605 GG vs GT+TT		rs1104760 AG+GG vs AA		rs2688513 AA vs AG+GG		rs2246901 AA vs AC+CC	
	AOR (95% CI)	*P*	AOR (95% CI)	*P*	AOR (95% CI)	*P*	AOR (95% CI)	*P*
Age								
< 61 years	1.132 (0.567–2.256)	0.726	1.715 (0.877–3.354)	0.115	0.869 (0.442–1.709)	0.683	0.993 (0.504–1.957)	0.983
≥ 61 years	0.948 (0.497–1.810)	0.872	2.382 (1.260–4.503)	**0.008**	0.808 (0.426–1.532)	0.514	0.980 (0.520–1.846)	0.950
Gender								
Male	0.882 (0.437–1.779)	0.726	2.210 (1.110–4.402)	**0.024**	0.739 (0.367–1.492)	0.399	0.825 (0.413–1.649)	0.586
Female	1.113 (0.587–2.112)	0.743	1.939 (1.041–3.614)	**0.037**	0.830 (0.446–1.544)	0.556	1.084 (0.581–2.023)	0.799
BMI								
< 25	1.252 (0.699–2.241)	0.449	1.448 (0.834–2.515)	0.188	1.207 (0.678–2.150)	0.523	1.140 (0.641–2.025)	0.656
≥ 25	0.778 (0.346–1.751)	0.544	3.999 (1.778–8.996)	**0.001**	0.393 (0.169–0.910)	**0.029**	0.803 (0.360–1.791)	0.592
HTN								
No	0.863 (0.418–1.782)	0.691	2.055 (1.014–4.163)	**0.046**	0.838 (0.407–1.724)	0.631	0.734 (0.360–1.496)	0.394
Yes	1.159 (0.613–2.193)	0.650	2.018 (1.084–3.756)	**0.027**	0.868 (0.465–1.618)	0.655	1.321 (0.704–2.478)	0.386
DM								
No	1.116 (0.656–1.899)	0.686	1.837 (1.099–3.070)	**0.020**	0.980 (0.581–1.655)	0.941	1.111 (0.659–1.873)	0.693
Yes	0.959 (0.346–2.655)	0.935	2.292 (0.801–6.557)	0.122	0.501 (0.179–1.405)	0.189	0.779 (0.283–2.140)	0.627
Smoking								
No	1.083 (0.566–2.075)	0.810	1.624 (0.869–3.035)	0.128	0.730 (0.387–1.377)	0.331	0.955 (0.508–1.795)	0.885
Yes	0.757 (0.329–1.742)	0.512	2.932 (1.259–6.827)	**0.013**	0.704 (0.304–1.633)	0.414	0.717 (0.310–1.663)	0.439
Hcy (μmol/L)								
< 13.3	1.176 (0.683–2.027)	0.559	2.429 (1.455–4.055)	**0.001**	0.765 (0.444–1.317)	0.333	1.019 (0.616–1.687)	0.942
≥ 13.3	1.907 (0.458–7.932)	0.375	1.269 (0.381–4.235)	0.698	2.023 (0.522–7.837)	0.308	2.525 (0.602–0.582)	0.205
Folate (nmol/L)								
> 3.7	0.991 (0.595–1.652)	0.974	2.429 (1.455–4.055)	**0.001**	0.761 (0.459–1.263)	0.290	1.019 (0.616–1.687)	0.942
≤ 3.7	4.090 (0.661–5.297)	0.130	0.375 (0.070–2.014)	0.253	2.100 (0.435–0.143)	0.356	1.123 (0.261–4.838)	0.876
TG (mg/dL)								
< 150	1.601 (0.856–2.994)	0.141	1.903 (1.092–3.317)	**0.023**	1.281 (0.694–2.365)	0.429	1.601 (0.856–2.994)	0.141
≥ 150	1.099 (0.429–2.818)	0.844	1.651 (0.693–3.935)	0.258	0.738 (0.285–1.912)	0.532	0.755 (0.307–1.858)	0.541
HDL-C (mg/dL)								
≥ 40(M), 50(F)	0.929 (0.461–1.873)	0.837	2.612 (1.322–5.162)	**0.006**	0.740 (0.371–1.475)	0.392	0.879 (0.441–1.754)	0.715
< 40(M), 50(F)	1.129 (0.599–2.127)	0.708	1.671 (0.897–3.114)	0.106	0.911 (0.489–1.697)	0.768	1.120 (0.600–2.092)	0.722
LDL-C (mg/dL)								
< 130	0.846 (0.392–1.826)	0.670	2.697 (1.302–5.585)	**0.008**	0.666 (0.318–1.394)	0.281	0.697 (0.333–1.459)	0.338
≥ 130	0.371 (0.055–2.497)	0.308	11.116 (1.357–1.092)	**0.025**	0.331 (0.047–2.334)	0.267	0.506 (0.068–3.790)	0.507

CRC, colorectal cancer; BMI, body mass index; HTN, hypertension; DM, diabetes mellitus; Hcy, plasma homocysteine; TG, triglyceride; HDL-C, high-density lipoprotein cholesterol; LDL-C, low-density lipoprotein cholesterol; T.chol, total cholesterol.

AOR is adjusted by age, sex, hypertension, diabetes mellitus, body mass index, high-density lipoprotein cholesterol.

Upper and lower 15% cut-off values of homocysteine and folate were 13.3 μmol/L and 3.7 ng/mL, respectively.

### Combined effects of *MUC4* polymorphisms on the occurrence of CRC

To identify the combined effects of four *MUC4* polymorphisms on CRC susceptibility, we analyzed haplotype and genotype combinations. The G-G-A-A assembly (*MUC4* rs882605 G>T/rs1104760 A>G/rs2688513 A>G/rs2246901 A>C) was associated with a decreased CRC prevalence compared to the reference assembly (AOR = 0.286, 95% CI: 0.151–0.539, *P* < 0.0001, FDR*-P* = 0.001), and its several subset combinations were also associated with decreased CRC occurrence compared to each reference assembly (Table 5, S2 Table). Interestingly, among the subsets, combinations that include the rs1104760 G allele had a significant impact on CRC risk (rs882605 G/rs1104760 G/rs2688513 A, OR = 0.313, *P* < 0.0001, FDR*-P* = 0.001; rs882605 G/rs1104760 G/rs2246901 A, OR = 0.285, *P* < 0.0001, FDR*-P* = 0.001; rs1104760 G/rs2688513 A/rs2246901 A, OR = 0.309, *P* < 0.0001, FDR*-P* = 0.001; rs882605 G/rs1104760 G, OR = 0.370, *P* < 0.0001, FDR*-P* = 0.0003; rs1104760 G/rs2688513 A, OR = 0.369, *P* = 0.0001; FDR*-P* = 0.0003; rs1104760 G/rs2246901 A, OR = 0.316, *P* < 0.0001, FDR*-P* = 0.0003) when setting a combination of each major alleles as a reference. Additionally, the combination of the *MUC4* rs882605 T allele and rs1104760 A allele, which is not a subset of the G-G-A-A assembly, was associated with decreased CRC prevalence (OR = 0.354, 95% CI: 0.146–0.858, *P* < 0.016, FDR*-P* = 0.024). All significant allele combinations maintained significant *P-*values after the FDR*-P* test.

**Table 5:** Haplotype analysis of four *MUC4* polymorphisms between controls and CRC patients.

Haplotype	Controls (n = 840)	CRC (n = 928)	OR (95% CI)	*P*	FDR*-P*
*MUC4* rs882605 G>T/rs1104760 A>G/ rs2688513 A>G/rs2246901 A>C					
G-A-A-A	680 (81.0)	794 (85.6)	1.000(reference)		
G-A-A-C	7 (0.8)	7 (0.8)	0.856 (0.299–2.454)	0.773	0.892
G-A-G-A	1 (0.1)	2 (0.2)	1.713 (0.155–18.94)	1.000	1.000
G-A-G-C	6 (0.7)	4 (0.4)	0.571 (0.160–2.032)	0.528	0.816
G-G-A-A	39 (4.6)	13 (1.4)	0.286 (0.151–0.539)	**<0.0001**	**0.001**
G-G-A-C	2 (0.2)	1 (0.1)	0.428 (0.039–4.736)	0.598	0.816
G-G-G-A	3 (0.4)	1 (0.1)	0.286 (0.030–2.752)	0.341	0.816
G-G-G-C	7 (0.8)	6 (0.6)	0.734 (0.246–2.195)	0.579	0.816
T-A-A-A	11 (1.3)	6 (0.6)	0.467 (0.172–1.270)	0.127	0.639
T-A-A-C	2 (0.2)	0 (0.0)	0.171 (0.008–3.577)	0.213	0.639
T-A-G-A	1 (0.1)	0 (0.0)	0.286 (0.012–7.025)	0.462	0.816
T-A-G-C	4 (0.5)	1 (0.1)	0.214 (0.024–1.921)	0.188	0.639
T-G-A-A	2 (0.2)	1 (0.1)	0.428 (0.039–4.736)	0.598	0.816
T-G-A-C	3 (0.4)	3 (0.3)	0.856 (0.172–4.259)	1.000	1.000
T-G-G-A	7 (0.8)	3 (0.3)	0.367 (0.095–1.425)	0.202	0.639
T-G-G-C	67 (8.0)	84 (9.1)	1.074 (0.767–1.504)	0.679	0.849

CRC, colorectal cancer; OR, odds ratio; 95% CI, 95% confidence interval.

Genotype combination analysis showed a similar pattern to that of haplotype analysis (S2 and S3 Tables). Most combinations that showed significant associations with decreased CRC risk were composed of the G-G-A-A allele assembly, which showed a significant effect (Table 5). The two combinations that did not contain significant alleles were associated only with reduced susceptibility to colon cancer. Interestingly, the common genotypes of the two combinations included the AA genotype of rs1104760, including AA/AG of rs1104760 and rs2688513 (AOR = 0.111, 95% CI: 0.016–0.763, *P* = 0.025, FDR*-P* = 0.040) and AA/AC of rs1104760 and rs2246901 (AOR = 0.149, 95% CI: 0.033–0.675, *P* = 0.014, FDR*-P* = 0.037).

Additionally, linkage disequilibrium (LD) block analysis was conducted to measure LD between polymorphisms (S2 Fig). Strong linkage disequilibrium was observed between each single nucleotide polymorphism (SNP), and the strongest correlation was found between *MUC4* rs2246901 A>C and rs2688513 A>G (R^2^ = 0.88), but an LD block was not found.

### *MUC4* rs2688513 A>G is associated with a poor CRC prognosis

**Fig 3:**
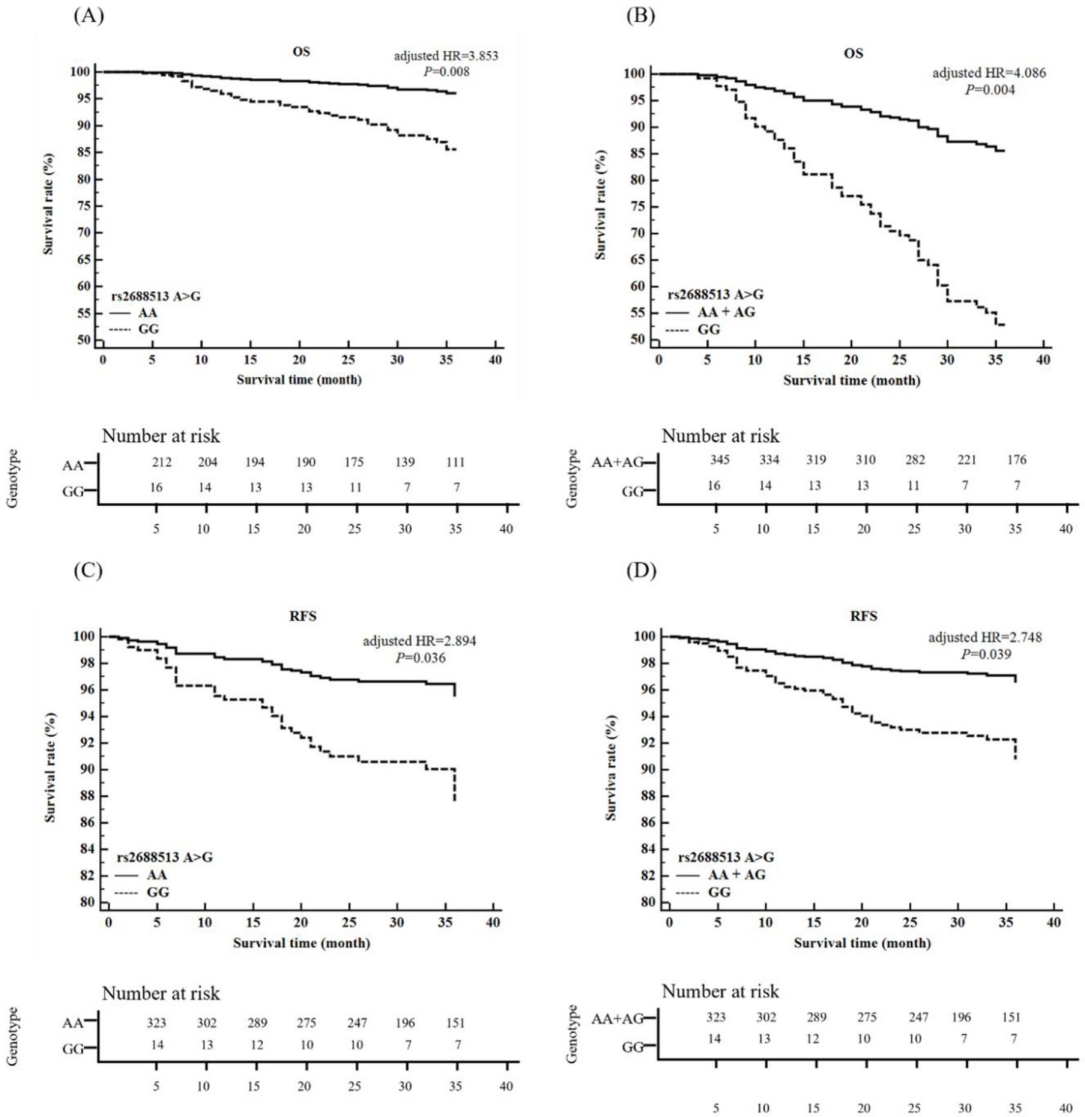
Overall survival (OS) plot and relapse-free survival (RFS) plot of *MUC4* rs2688513A>G polymorphisms in colorectal cancer according to the Cox proportional hazards model. *MUC4* rs2688513 GG genotype was associated with increased mortality (A, B) and increased colorectal cancer relapse (C, D) compared to the AA genotype and AA+AG model at 3 years.

We examined the effect of *MUC4* polymorphisms on CRC prognosis with regard to the times until mortality and relapse. For both 3- and 5-year mortality, the *MUC4* rs2688513 GG genotype showed a significant association with an increased HR of CRC compared to the AA genotype and AA+AG model (Fig 3(A), 3(B), S4 and S6 Tables). In addition, in rectal cancer patients, the GG genotype also showed a correlation with an increased death rate compared to the AA genotype and AA+AG model at 3 years (GG genotype, adjusted HR = 10.341, 95% CI, 1.396–6.586, *P* = 0.023; recessive model, adjusted HR = 7.884, 95% CI, 1.488–1.772, *P* = 0.016) and 5 years (GG genotype, adjusted HR = 6.496, 95% CI: 1.097–8.466, *P* = 0.040; recessive model, adjusted HR = 5.848, 95% CI, 1.175–9.111, *P* = 0.032). In colon cancer, the GG genotype remained significant for 3-year mortality compared to the AA genotype (adjusted HR = 4.099, 95% CI: 1.075–5.633, *P* = 0.040). Interestingly, *MUC4* rs1104760 recessive model showed a strong effect on increased mortality of colon cancer at both 3 and 5 years (3 years, adjusted HR = 3.703, 95% CI: 1.280–0.712, *P* = 0.016; 5 years, adjusted HR = 3.474, 95% CI: 1.244–9.706, *P* = 0.018) but showed no association with the risk of rectal cancer. Regarding relapse, the *MUC4* rs2688513 GG genotype was associated with a high level of CRC relapse-free survival compared to the AA genotype and AA+AG model at both 3 years (GG genotype, adjusted HR = 2.894, 95% CI: 1.078–7.768, *P* = 0.036; recessive model, adjusted HR = 2.748, 95% CI: 1.059–7.130, *P* = 0.039) and 5 years (GG genotype, adjusted HR = 0.522, 95% CI: 1.068–7.671, *P* = 0.038; recessive model, adjusted HR = 2.737, 95% CI: 1.055–7.100, *P* = 0.040) (Fig 3, S5 and S7 Tables).

### Discussion

CRC is closely associated with various genetic conditions related to alterations in intestinal homeostasis that allow its carcinogenesis to proceed [22]. Since the accumulation of genetic mutations can lead to cancer development, several genetic polymorphisms were correlated with CRC risk in genome-wide association studies [23, 24]. One representative CRC-related gene, *MUC4*, has been reported to show aberrant expression in CRC patients, but only one study suggested a significant role of *MUC4* SNPs in CRC progression [13]. In this study, we analyzed the associations between *MUC4* rs882605 G>T, rs1104760 A>G, rs2688513 A>G, and rs2246901 A>C polymorphisms and CRC prevalence and prognosis to elucidate the multifunctional aspects of *MUC4* genetic polymorphisms.

CRC development is associated with both genetic and environmental factors. In particular, obesity is firmly established as a significant risk factor for CRC development, and dyslipidemia is a well-known obesity-related metabolic feature [5]. The role of lipid alterations in CRC etiology is not clear but HDL-C and LDL-C levels are considered to be correlated with CRC development. HDL-C has anti-inflammatory and immunomodulatory activities and reduces the risk of CRC [25]. This lipoprotein prevents the conversion of macrophages to the pro-inflammatory M1 phenotype, thus decreasing the pro-inflammatory milieu, which can lead to cancer by increasing the irregularities in the intestine [26]. In contrast, LDL-C is suggested to induce inflammation which can increase the risk of CRC, although the mechanism remains unclear. LDL-C is hypothesized to promote cholesterol accumulation and enhance inflammation, inducing atherosclerosis, which is highly associated with CRC and shares common risk factors [27–29]. In addition, increased metastasis was associated with a high LDL-C level [30]. Mucin glycoproteins may be correlated with lipoproteins through their involvement in inflammation. As the major macromolecular components of mucus, mucin glycoproteins can regulate inflammation in the intestine, which can damage the mucus barrier, worsen mucus quality, and reduce mucus production [31, 32]. Consistent with previous results, our results showed a high correlation between the *MUC4* rs1104760 A>G polymorphism and lipoproteins. *MUC4* rs1104760 AA variant had a synergistic effect with LDL-C levels, exhibiting five-fold higher CRC risk when combined with LDL-C levels in the risk range compared with AG+GG variants in individuals with LDL-C levels in the normal range (Table 4). Moreover, the *MUC4* rs1104760 AA variant exhibited an approximately four-fold increased risk of CRC when combined with HDL-C levels in the risk range compared with the AG+GG variant in individuals with HDL-C levels in the normal range (Fig 1). Furthermore, ANOVA showed that the *MUC4* rs1104760 AA variant was associated with significantly higher LDL-C levels than those found with the AG and GG variants (S1 Fig). As a result, we suggest that *MUC4* rs1104760 A>G is a functional SNP in *MUC4*, and its AA genotype affects LDL-C levels and inflammation, inducing CRC development.

The *MUC4* rs1104760 A>G polymorphism was associated with CRC occurrence without combination with metabolic factors. In the genetic association analysis, its G allele had a protective tendency for CRC risk compared with that of the A allele, which was associated with high LDL-C levels. Consistent with previous studies of reduced *MUC4* expression in CRC patients [33, 34], our results elucidated a protective role of *MUC4* in CRC patients according to its SNPs. Furthermore, we found that the *MUC4* rs1104760 A>G variant combined with LDL-C levels in the risk range showed greater predictive value for CRC occurrence than the same variant combined with LDL-C levels in the control range through ROC curve analysis, the representative diagnostic test (Fig 2). However, the exact mechanism by which the *MUC4* rs1104760 A>G affects inflammation and LDL-C levels and contributes to CRC development should be further studied.

Interestingly, we also showed an aggressive role of *MUC4* regarding CRC prognosis, which helped to elucidate the controversial role of *MUC4* in CRC patients. In the survival analysis, the *MUC4* rs2688513 GG variant was associated with a poor prognosis of CRC compared with the AA and AA +AG variants (Fig 3). This result is consistent with previous findings indicating that *MUC4* was overexpressed in a subset of CRC patients with a worse prognosis [11, 12]. *MUC4* contains three epidermal growth factor (EGF) domains, which is a common mitogenic factor that stimulates the proliferation of different cell types, especially fibroblasts and epithelial cells [30]. *MUC4* may act as an intramembrane ligand for the receptor tyrosine kinase ErbB2 and perform an anti-apoptotic function to promote tumor progression [13, 34, 35]. Additionally, we showed that the G allele was not associated with decreased CRC risk in the haplotype combination analysis, while its A allele was significantly associated with decreased CRC risk when combined with the A allele of the *MUC4* rs1104760 polymorphism (Table 5, S2 Table). Therefore, we suggest that *MUC4* has a significant effect on worsening the prognosis of CRC when the GG genotype of *MUC4* 2688513 polymorphism is present.

As missense variants located on the second exon, *MUC4* rs1104760 A>G and 2688513 A>G polymorphisms change the second and first base of the codon, converting isoleucine (ATC) to threonine (ACC) and serine (TCA) to proline (CCA), respectively [16]. Thus, both polymorphisms are highly likely to alter the gene function and be significantly associated with the prevalence and prognosis of CRC. Our results are consistent with those of previous studies in which underexpression of *MUC4* was reported in most CRC patients while overexpression of *MUC4* was reported in a subset of CRC patients with a poor prognosis. Therefore, we suggest the *MUC4* rs1104760 A>G polymorphism as a novel biomarker for CRC treatment because it is correlated with LDL-C levels and may affect inflammation in the intestine, thus inducing CRC development. Additionally, we suggest the *MUC4* 2688513 A>G polymorphism as a prognostic marker of a poor CRC prognosis although further research is needed to determine the correlation between the *MUC4* 2688513 A>G polymorphism and EGF domains.

In the medical treatment of CRC, a trend shifting from surgery as the main mode of treatment to personalized treatments for individual care has developed since CRC is a complicated disease that occurs with the accumulation of genetic mutations and changes in epigenetic factors [36]. Genetic variants can be useful for personalized cancer treatment by predicting the impact of each allele on disease development or prognosis. To our knowledge, this study is the first to suggest *MUC4* polymorphisms as possible biomarkers for CRC risk while considering related metabolic factors. *MUC4* rs1104760 A>G may be a predictor for individual susceptibility to CRC, and *MUC4* rs2688513 A>G may be a prognostic marker. By applying these concepts to clinical measures, it will be possible to distinguish whether patients require stronger preventative measures for CRC. However, there are some limitations to our study. First, the exact mechanisms of *MUC4* were not confirmed. Although the present study showed a statistically significant association between LDL-C and *MUC4* rs1104760 A>G, a clear explanation was not available. However, *MUC4* rs1104760 A>G has a strong potential for use as a biomarker because a sensitivity analysis (ROC analysis) indicated its significance in predicting CRC. Second, the study subjects were limited to a small sample size recruited in one hospital but our studies satisfied HWE. Further studies should include more patients to establish *MUC4* SNPs as biomarkers.

## Conclusion

We investigated *MUC4* rs882605 G>T, rs1104760 A>G, rs2688513 A>G, and rs2246901 A>C variants in controls and CRC patients and showed their association with susceptibility to and prognosis of CRC. In particular, *MUC4* rs1104760 mutant allele had a protective effect against CRC prevalence compared to the wild allele. Furthermore, *MUC4* rs1104760 A>G had a strong correlation with LDL-C with regard to CRC risk and had a predictive value in CRC patients with LDL-C levels in the risk range. Based on the effect of LDL-C on inflammation, which leads to CRC development, we suggest that the *MUC4* rs1104760 A>G plays a substantial role in CRC pathology via the inflammatory processes related to LDL-C. In addition, as the *MUC4* rs2688513 mutant genotype was associated with a worse prognosis of CRC compared with the wild genotype, we suggest that the *MUC4* rs2688513 A>G polymorphism is a prospective marker for CRC progression. This is the first study to elucidate the multifunctional role of *MUC4* in CRC patients while considering metabolic factors. Regarding the recent medical focus on personalized treatment, our results provide a significant cornerstone for further studies aimed to utilize *MUC4* polymorphisms as individualized factors for CRC treatment and early diagnosis.

## Supporting information

S1 Fig Association between LDL-C level and *MUC4* rs1104760 A>G and *MUC4* rs2688513 A>G. In both *MUC4* rs1104760 A>G and *MUC4* rs2688513 A>G, wild genotypes have a higher mean of LDL-C concentration, and mutant genotypes have a lower mean of LDL-C concentration. All p-values were statistically significant (rs1104760 A>G, *P* = 0.037; rs2688513 A>G, *P* = 0.027).
(DOCX)

S2 Fig Linkage disequilibrium plots for four SNPs of the *MUC4* gene using Haploview software. LD was calculated using D’ and R^2^ values by performing the Haploview software which shows linkage disequilibrium between the SNPs in the LD plot. D is the coefficient of LD and R^2^ is the squared correlation. The number in the block denotes LD calculated using R^2^; a higher number means high LD. The colored squares show the strength of LD; red means high LD, pink means moderate LD, and white means low LD. However, the combination specified by the block was not found.
(DOCX)

S1 Table CRC prevalence by interaction analysis between four *MUC4* genotypes and environmental factors. (DOCX)

S2 Table Haplotype analysis of *MUC4* polymorphisms in controls and CRC patients. (DOCX)

S3 Table Genotype combination analysis of *MUC4* polymorphisms in controls and colorectal, colon and rectum cancer patients. (DOCX)

S4 Table *MUC4* genotype frequencies and patient 3-year mortality in overall, colon, and rectum cancer. (DOCX)

S5 Table *MUC4* polymorphism genotype frequencies and patient 3-year relapse in overall, colon, and rectum cancer. (DOCX)

S6 Table *MUC4* polymorphism genotype frequencies and patient 5-year mortality in overall, colon, and rectum cancer. (DOCX)

S7 Table *MUC4* polymorphism genotype frequencies and patient 5-year relapse in overall, colon, and rectum cancer. (DOCX)

S8 Table Statistical power of genetic association of less than 0.05 *p*-value in Table 2. (DOCX)
