# Supplementary material for: Genetic variants of MUC4 are associated with susceptibility to and mortality of colorectal cancer and exhibit synergistic effects with LDL-C levels
Source: PLoS One. 2023 Jun 29;18(6):e0287768. doi: 10.1371/journal.pone.0287768 (PMC10310026; doi:10.1371/journal.pone.0287768)
Supplement: S1 Fig — In both MUC4 rs1104760 A>G and MUC4 rs2688513 A>G, wild genotypes have a higher mean of LDL-C concentration, and mutant genotypes have a lower mean of LDL-C concentration. All p-values were statistically significant (rs1104760 A>G, P = 0.037; rs2688513 A>G, P = 0.027). (DOCX) [file pone.0287768.s001.docx]

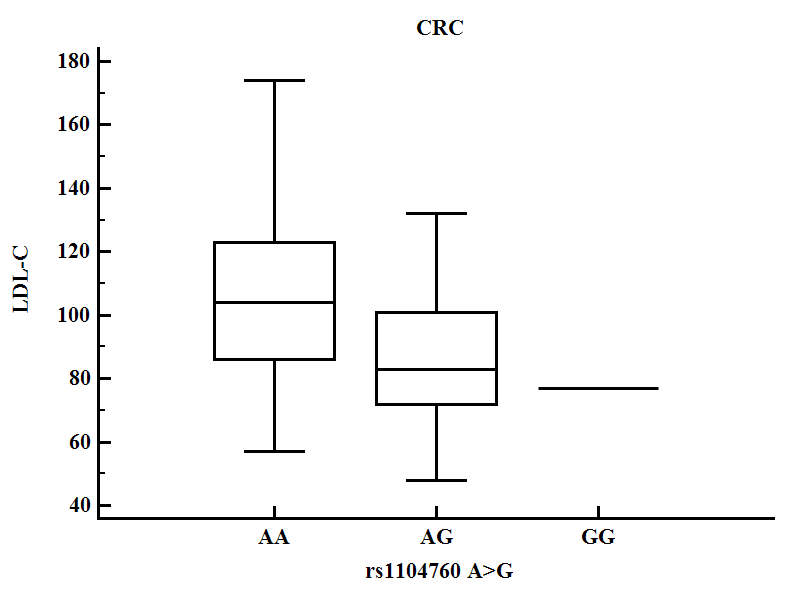

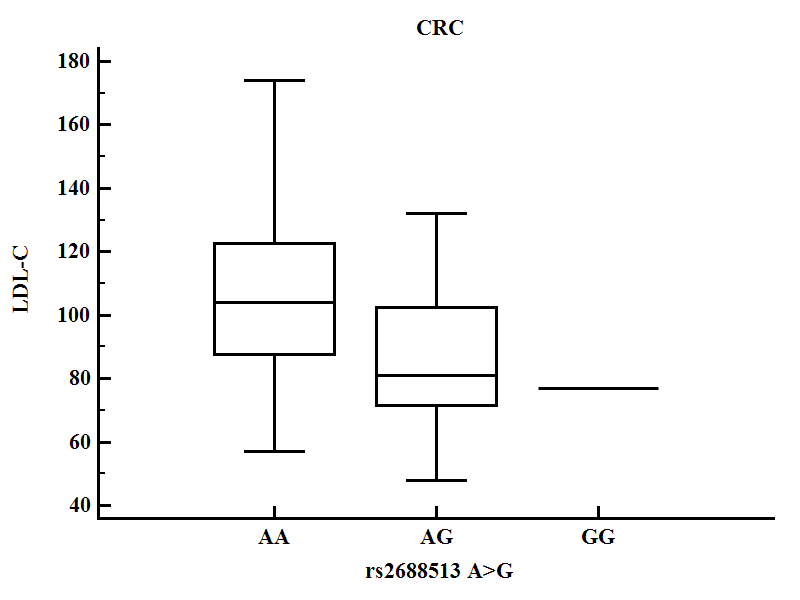


**S1 Figure. Association between LDL-C level and** ***MUC4* rs1104760 A>G and *MUC4* rs2688513 A>G.** In both *MUC4* rs1104760 A>G and *MUC4* rs2688513 A>G, wild genotypes have a higher mean LDL-C concentration, and mutant genotypes have a lower mean LDL-C concentration. All *p*-values were statistically significant (rs1104760 A>G, *P* = 0.037; rs2688513 A>G, *P* = 0.027).
