# Supplementary material for: Genetic variants of MUC4 are associated with susceptibility to and mortality of colorectal cancer and exhibit synergistic effects with LDL-C levels
Source: PLoS One. 2023 Jun 29;18(6):e0287768. doi: 10.1371/journal.pone.0287768 (PMC10310026; doi:10.1371/journal.pone.0287768)
Supplement: S2 Fig — LD was calculated using D’ and R2 values by performing the Haploview software which shows linkage disequilibrium between the SNPs in the LD plot. D is the coefficient of LD and R2 is the squared correlation. The number in the block denotes LD calculated using R2; a higher number means high LD. The colored squares show the strength of LD; red means high LD, pink means moderate LD, and white means low LD. However, the combination specified by the block was not found. (DOCX) [file pone.0287768.s002.docx]

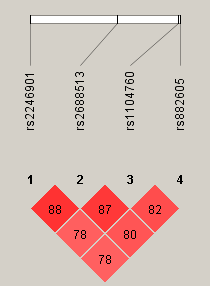


**S2 Figure. Linkage disequilibrium plots for four SNPs of the *MUC4* gene** **using Haploview software.** LD was calculated using D' and R^2^ values by performing the Haploview software which shows linkage disequilibrium between the SNPs in the LD plot. D is the coefficient of LD and R^2^ is the squared correlation. The number in the block denotes LD calculated using R^2^; a higher number means high LD. The colored squares show the strength of LD; red means high LD, pink means moderate LD, and white means low LD. However, the combination specified by the block was not found.
