# Supplementary material for: Genetic variants of MUC4 are associated with susceptibility to and mortality of colorectal cancer and exhibit synergistic effects with LDL-C levels
Source: PLoS One. 2023 Jun 29;18(6):e0287768. doi: 10.1371/journal.pone.0287768 (PMC10310026; doi:10.1371/journal.pone.0287768)
Supplement: S1 Table — (DOCX) [file pone.0287768.s003.docx]

| **S1 Table. CRC prevalence by interaction analysis between four *MUC4* genotypes and environmental factors** | | | | | | | | |
| --- | --- | --- | --- | --- | --- | --- | --- | --- |
| Characteristics | rs882605 GG | rs882605  GT+TT | rs1104760 AG+GG | rs1104760  AA | rs2688513  AA | rs2688513 AG+GG | rs2246901  AA | rs2246901 AC+CC |
|  | AOR (95% CI) | AOR (95% CI) | AOR (95% CI) | AOR (95% CI) | AOR (95% CI) | AOR (95% CI) | AOR (95% CI) | AOR (95% CI) |
| Age |  |  |  |  |  |  |  |  |
| < 61 years | 1.000 (reference) | 1.132 (0.567-2.256) | 1.000 (reference) | 1.715 (0.877-3.354) | 1.000 (reference) | 0.869 (0.442-1.709) | 1.000 (reference) | 0.993 (0.504-1.957) |
| ≥ 61 years | 0.923 (0.502-1.697) | 0.993 (0.495-1.995) | 0.755 (0.385-1.48) | 1.645 (0.826-3.275) | 0.796 (0.417-1.521) | 0.694 (0.339-1.422) | 0.900 (0.478-1.693) | 0.881 (0.445-1.746) |
| Gender |  |  |  |  |  |  |  |  |
| Male | 1.000 (reference) | 0.882 (0.437-1.779) | 1.000 (reference) | **2.210 (1.11-4.402)** | 1.000 (reference) | 0.739 (0.367-1.492) | 1.000 (reference) | 0.825 (0.413-1.649) |
| Female | 1.107 (0.622-1.970) | 1.247 (0.639-2.433) | 1.190 (0.624-2.272) | **2.522 (1.28-4.969)** | 1.197 (0.652-2.197) | 0.960 (0.515-1.790) | 1.103 (0.607-2.005) | 1.129 (0.603-2.116) |
| BMI |  |  |  |  |  |  |  |  |
| < 25 | 1.000 (reference) | 1.213 (0.684-2.153) | 1.000 (reference) | 1.448 (0.834-2.515) | 1.000 (reference) | 1.179 (0.668-2.078) | 1.000 (reference) | 1.147 (0.652-2.018) |
| ≥ 25 | 0.630 (0.352-1.126) | 0.437 (0.203-0.941) | **0.359 (0.184-0.701)** | 1.491 (0.736-3.02) | 0.813 (0.438-1.509) | **0.314 (0.152-0.648)** | 0.649 (0.353-1.191) | 0.446 (0.218-0.915) |
| HTN |  |  |  |  |  |  |  |  |
| No | 1.000 (reference) | 0.863 (0.418-1.782) | 1.000 (reference) | **2.055 (1.014-4.163)** | 1.000 (reference) | 0.838 (0.407-1.724) | 1.000 (reference) | 0.734 (0.36-1.496) |
| Yes | 1.738 (0.932-3.242) | 2.017 (0.971-4.193) | **2.062 (1.009-4.217)** | **3.894 (1.911-7.937)** | **2.114 (1.084-4.119)** | 1.518 (0.760-3.032) | 1.529 (0.794-2.943) | 1.922 (0.923-4.004) |
| DM |  |  |  |  |  |  |  |  |
| No | 1.000 (reference) | 1.116 (0.656-1.899) | 1.000 (reference) | **1.837 (1.099-3.07)** | 1.000 (reference) | 0.980 (0.581-1.655) | 1.000 (reference) | 1.111 (0.659-1.873) |
| Yes | 4.009 (1.881-8.545) | 2.969 (1.274-6.921) | **2.726 (1.285-5.784)** | **8.130 (3.268-0.226)** | **5.618 (2.378-3.272)** | 2.103 (0.974-4.540) | **4.502 (2.046-9.903)** | **2.731 (1.199-6.218)** |
| Smoking |  |  |  |  |  |  |  |  |
| No | 1.000 (reference) | 1.080 (0.563-2.069) | 1.000 (reference) | 1.640 (0.878-3.064) | 1.000 (reference) | 0.727 (0.385-1.370) | 1.000 (reference) | 0.950 (0.505-1.788) |
| Yes | **0.158 (0.075-0.333)** | **0.159 (0.065-0.393)** | **0.107 (0.043-0.266)** | **0.291 (0.123-0.688)** | **0.146 (0.068-0.313)** | **0.118 (0.046-0.300)** | **0.151 (0.070-0.323)** | **0.113 (0.043-0.298)** |
| Hcy (μmol/L) |  |  |  |  |  |  |  |  |
| < 13.3 | 1.000 (reference) | 0.999 (0.598-1.669) | 1.000 (reference) | **2.396 (1.438-3.99)** | 1.000 (reference) | 0.715 (0.430-1.189) | 1.000 (reference) | 0.870 (0.525-1.443) |
| ≥ 13.3 | 0.943 (0.432-2.056) | 1.268 (0.395-4.071) | 1.376 (0.522-3.626) | 1.860 (0.794-4.355) | 0.821 (0.361-1.870) | 1.008 (0.339-2.999) | 0.864 (0.390-1.913) | 1.352 (0.426-4.296) |
| Folate (nmol/L) |  |  |  |  |  |  |  |  |
| > 3.7 | 1.000 (reference) | 0.991 (0.595-1.652) | 1.000 (reference) | **2.429 (1.455-4.055)** | 1.000 (reference) | 0.761 (0.459-1.263) | 1.000 (reference) | 1.019 (0.616-1.687) |
| ≤ 3.7 | 1.676 (0.787-3.569) | 5.001 (1.078-3.197) | **5.583 (1.509-0.658)** | **3.197 (1.466-6.97)** | 1.708 (0.778-3.750) | 3.079 (0.829-1.434) | 2.136 (0.964-4.733) | 2.318 (0.71-7.568) |
| TG (mg/dL) |  |  |  |  |  |  |  |  |
| < 150 | 1.000 (reference) | 1.275 (0.712-2.283) | 1.000 (reference) | **1.903 (1.092-3.317)** | 1.000 (reference) | 1.087 (0.612-1.928) | 1.000 (reference) | 1.391 (0.775-2.496) |
| ≥ 150 | 0.710 (0.371-1.360) | 0.486 (0.228-1.033) | 0.627 (0.303-1.298) | 1.450 (0.703-2.991) | 0.789 (0.397-1.570) | **0.397 (0.195-0.808)** | 0.847 (0.424-1.692) | **0.508 (0.259-0.998)** |
| HDL-C (mg/dL) |  |  |  |  |  |  |  |  |
| ≥ 40 (M), 50 (F) | 1.000 (reference) | 0.834 (0.526-1.322) | 1.000 (reference) | **1.661 (1.061-2.6)** | 1.000 (reference) | 0.787 (0.498-1.246) | 1.000 (reference) | 0.978 (0.619-1.546) |
| < 40 (M), 50 (F) | **1.838 (1.124-3.005)** | **2.049 (1.150-3.652)** | **2.079 (1.202-3.598)** | **3.645 (2.06-6.45)** | **1.948 (1.169-3.244)** | 1.726 (0.996-2.992) | **1.960 (1.183-3.249)** | **2.124 (1.22-3.696)** |
| LDL-C (mg/dL) |  |  |  |  |  |  |  |  |
| < 130 | 1.000 (reference) | 0.846 (0.392-1.826) | 1.000 (reference) | **2.697 (1.302-5.585)** | 1.000 (reference) | 0.666 (0.318-1.394) | 1.000 (reference) | 0.697 (0.333-1.459) |
| ≥ 130 | 0.444 (0.162-1.216) | 0.116 (0.027-0.497) | **0.223 (0.055-0.912)** | 1.179 (0.389-3.574) | 0.412 (0.146-1.166) | **0.085 (0.018-0.401)** | **0.355 (0.126-0.995)** | **0.108 (0.023-0.519)** |
| CRC, colorectal cancer; BMI, body mass index; HTN, hypertension; DM, diabetes mellitus; Hcy, plasma homocysteine; TG, triglyceride; HDL-C, high density lipoprotein cholesterol; LDL-C, low density lipoprotein cholesterol; T.chol, total cholesterol. AOR is adjusted by age, sex, hypertension, diabetes mellitus, body mass index, high density lipoprotein cholesterol. Upper and lower 15% cut-off values of homocysteine and folate were 13.3 μmol/L and 3.7 ng/mL, respectively. | | | | | | | | |
