# Supplementary material for: Genetic variants of MUC4 are associated with susceptibility to and mortality of colorectal cancer and exhibit synergistic effects with LDL-C levels
Source: PLoS One. 2023 Jun 29;18(6):e0287768. doi: 10.1371/journal.pone.0287768 (PMC10310026; doi:10.1371/journal.pone.0287768)
Supplement: S2 Table — (DOCX) [file pone.0287768.s004.docx]

| **S2 Table. Haplotype analysis of *MUC4* polymorphisms in controls and CRC patients** | | | | | |
| --- | --- | --- | --- | --- | --- |
| Haplotype | Controls  (n=840) | CRC (n=928) | OR (95% CI) | *P* | FDR*-P* |
| *MUC4* rs882605 G>T/rs1104760 A>G/rs2688513 A>G |  |  |  |  |  |
| G-A-A | 686 (163.3) | 801 (172.6) | 1.000(reference) |  |  |
| G-A-G | 7 (1.7) | 6 (1.3) | 0.734 (0.246-2.195) | 0.579 | 0.676 |
| G-G-A | 41 (9.8) | 15 (3.2) | 0.313 (0.172-0.571) | **<0.0001** | **0.001** |
| G-G-G | 9 (2.1) | 7 (1.5) | 0.666 (0.247-1.798) | 0.420 | 0.676 |
| T-A-A | 13 (3.1) | 6 (1.3) | 0.395 (0.149-1.046) | 0.053 | 0.186 |
| T-A-G | 4 (1.0) | 1 (0.2) | 0.214 (0.024-1.921) | 0.188 | 0.439 |
| T-G-A | 6 (1.4) | 5 (1.1) | 0.714 (0.217-2.349) | 0.577 | 0.676 |
| T-G-G | 74 (17.6) | 87 (18.8) | 1.007 (0.727-1.395) | 0.967 | 0.967 |
| *MUC4* rs882605 G>T/rs1104760 A>G/rs2246901 A>C |  |  |  |  |  |
| G-A-A | 680 (161.9) | 795 (171.3) | 1.000(reference) |  |  |
| G-A-C | 13 (3.1) | 11 (2.4) | 0.724 (0.322-1.626) | 0.432 | 0.504 |
| G-G-A | 42 (10.0) | 14 (3.0) | 0.285 (0.154-0.527) | **<0.0001** | **0.001** |
| G-G-C | 9 (2.1) | 7 (1.5) | 0.665 (0.246-1.796) | 0.418 | 0.504 |
| T-A-A | 12 (2.9) | 6 (1.3) | 0.428 (0.160-1.146) | 0.082 | 0.179 |
| T-A-C | 5 (1.2) | 1 (0.2) | 0.171 (0.020-1.469) | 0.102 | 0.179 |
| T-G-A | 10 (2.4) | 4 (0.9) | 0.342 (0.107-1.096) | 0.065 | 0.179 |
| T-G-C | 70 (16.7) | 88 (19.0) | 1.075 (0.773-1.496) | 0.667 | 0.667 |
| *MUC4* rs882605 G>T/rs2688513 A>G/rs2246901 A>C |  |  |  |  |  |
| G-A-A | 719 (171.2) | 807 (173.9) | 1.000(reference) |  |  |
| G-A-C | 9 (2.1) | 9 (1.9) | 0.891 (0.352-2.257) | 0.808 | 0.943 |
| G-G-A | 3 (0.7) | 3 (0.6) | 0.891 (0.179-4.430) | 1.000 | 1.000 |
| G-G-C | 13 (3.1) | 10 (2.2) | 0.685 (0.299-1.573) | 0.370 | 0.863 |
| T-A-A | 13 (3.1) | 7 (1.5) | 0.480 (0.190-1.209) | 0.112 | 0.452 |
| T-A-C | 5 (1.2) | 4 (0.9) | 0.713 (0.191-2.665) | 0.743 | 0.943 |
| T-G-A | 8 (1.9) | 3 (0.6) | 0.334 (0.088-1.265) | 0.129 | 0.452 |
| T-G-C | 70 (16.7) | 85 (18.3) | 1.082 (0.776-1.508) | 0.642 | 0.943 |
| *MUC4* rs1104760 A>G/rs2688513 A>G/rs2246901 A>C |  |  |  |  |  |
| A-A-A | 691 (164.5) | 800 (172.4) | 1.000(reference) |  |  |
| A-A-C | 8 (1.9) | 8 (1.7) | 0.864 (0.322-2.314) | 0.771 | 0.900 |
| A-G-A | 1 (0.2) | 2 (0.4) | 1.728 (0.156-19.100) | 1.000 | 1.000 |
| A-G-C | 10 (2.4) | 5 (1.1) | 0.432 (0.147-1.270) | 0.116 | 0.271 |
| G-A-A | 42 (10.0) | 15 (3.2) | 0.309 (0.170-0.561) | **<0.0001** | **0.001** |
| G-A-C | 6 (1.4) | 5 (1.1) | 0.720 (0.219-2.369) | 0.587 | 0.900 |
| G-G-A | 10 (2.4) | 4 (0.9) | 0.346 (0.108-1.107) | 0.103 | 0.271 |
| G-G-C | 73 (17.4) | 90 (19.4) | 1.065 (0.769-1.474) | 0.705 | 0.900 |
| *MUC4* rs882605 G>T/rs1104760 A>G |  |  |  |  |  |
| G-A | 693 (165.0) | 807 (173.9) | 1.000(reference) |  |  |
| G-G | 51 (12.1) | 22 (4.7) | 0.370 (0.222-0.617) | **<0.0001** | **0.0003** |
| T-A | 17 (4.0) | 7 (1.5) | 0.354 (0.146-0.858) | **0.016** | **0.024** |
| T-G | 80 (19.0) | 92 (19.8) | 0.988 (0.720-1.355) | 0.938 | 0.938 |
| *MUC4* rs882605 G>T/rs2688513 A>G |  |  |  |  |  |
| G-A | 728 (173.3) | 816 (175.9) | 1.000(reference) |  |  |
| G-G | 16 (3.8) | 13 (2.8) | 0.725 (0.346-1.517) | 0.3913 | 0.587 |
| T-A | 18 (4.3) | 11 (2.4) | 0.545 (0.256-1.162) | 0.111 | 0.333 |
| T-G | 78 (18.6) | 88 (19.0) | 1.007 (0.730-1.387) | 0.968 | 0.968 |
| *MUC4* rs882605 G>T/rs2246901 A>C |  |  |  |  |  |
| G-A | 722 (171.9) | 810 (174.6) | 1.000(reference) |  |  |
| G-C | 22 (5.2) | 19 (4.1) | 0.829 (0.369-1.865) | 0.650 | 0.719 |
| T-A | 22 (5.2) | 10 (2.2) | 0.415 (0.149-1.150) | 0.081 | 0.243 |
| T-C | 75 (17.9) | 89 (19.2) | 1.079 (0.712-1.635) | 0.719 | 0.719 |
| *MUC4* rs1104760 A>G/rs2688513 A>G |  |  |  |  |  |
| A-A | 699 (166.4) | 807 (173.9) | 1.000(reference) |  |  |
| A-G | 11 (2.6) | 7 (1.5) | 0.551 (0.213-1.430) | 0.214 | 0.321 |
| G-A | 47 (11.2) | 20 (4.3) | 0.369 (0.216-0.628) | **0.0001** | **0.0003** |
| G-G | 83 (19.8) | 94 (20.3) | 0.981 (0.718-1.340) | 0.904 | 0.904 |
| *MUC4* rs1104760 A>G/rs2246901 A>C |  |  |  |  |  |
| A-A | 692 (164.8) | 801 (172.6) | 1.000(reference) |  |  |
| A-C | 18 (4.3) | 13 (2.8) | 0.624 (0.304-1.283) | 0.196 | 0.294 |
| G-A | 52 (12.4) | 19 (4.1) | 0.316 (0.185-0.539) | **<0.0001** | **0.0003** |
| G-C | 79 (18.8) | 95 (20.5) | 1.039 (0.758-1.424) | 0.813 | 0.813 |
| *MUC4* rs2688513 A>G/rs2246901 A>C |  |  |  |  |  |
| A-A | 732 (174.3) | 814 (175.4) | 1.000(reference) |  |  |
| A-C | 14 (3.3) | 13 (2.8) | 0.835 (0.390-1.789) | 0.642 | 0.856 |
| G-A | 11 (2.6) | 6 (1.3) | 0.491 (0.181-1.333) | 0.154 | 0.462 |
| G-C | 83 (19.8) | 95 (20.5) | 1.029 (0.754-1.405) | 0.856 | 0.856 |
| CRC, colorectal cancer; OR, odds ratio; 95% CI, 95% confidence interval. | | | | | |
