# Supplementary material for: Genetic variants of MUC4 are associated with susceptibility to and mortality of colorectal cancer and exhibit synergistic effects with LDL-C levels
Source: PLoS One. 2023 Jun 29;18(6):e0287768. doi: 10.1371/journal.pone.0287768 (PMC10310026; doi:10.1371/journal.pone.0287768)
Supplement: S3 Table — (DOCX) [file pone.0287768.s005.docx]

| **S3 Table. Genotype combination analysis of *MUC4* polymorphisms in controls and colorectal, colon and rectum cancer patients** | | | | | | | | | | | | | |
| --- | --- | --- | --- | --- | --- | --- | --- | --- | --- | --- | --- | --- | --- |
| Genotype | Controls (n=420) | CRC  patients (n=464) | AOR (95% CI) | *P* | FDR*-P* | Colon (n=260) | AOR (95% CI) | *P* | FDR*-P* | Rectum (n=192) | AOR (95% CI) | *P* | FDR*-P* |
| *MUC4* rs882605 G>T/rs1104760 A>G |  |  |  |  |  |  |  |  |  |  |  |  |  |
| GG/AA | 176 (58.7) | 248 (73.8) | 1.000(reference) |  |  | 144 (48.0) | 1.000(reference) |  |  | 102 (42.9) | 1.000(reference) |  |  |
| GG/AG | 62 (20.7) | 32 (9.5) | 0.122 (0.051-0.293) | **<0.0001** | **0.0002** | 12 (4.0) | 0.061 (0.016-0.234) | **<0.0001** | **0.0002** | 16 (6.7) | 0.228 (0.084-0.615) | **0.004** | **0.008** |
| GG/GG | 9 (3.0) | 3 (0.9) | 0.254 (0.035-1.862) | 0.177 | 0.283 | 2 (0.7) | N/A | 0.426 | 0.283 | 1 (0.4) | N/A |  |  |
| GT/AA | 23 (7.7) | 11 (3.3) | 0.985 (0.098-9.943) | 0.990 | 0.990 | 6 (2.0) | 0.498 (0.027-9.074) | 0.638 | 0.990 | 5 (2.1) | 1.934 (0.165-2.734) | 0.600 | 0.686 |
| GT/AG | 111 (37.0) | 147 (43.8) | 0.84 (0.493-1.430) | 0.520 | 0.594 | 80 (26.7) | 0.669 (0.368-1.219) | 0.190 | 0.594 | 62 (26.1) | 1.006 (0.537-1.884) | 0.986 | 0.986 |
| GT/GG | 19 (6.3) | 5 (1.5) | 0.194 (0.046-0.818) | **0.026** | 0.052 | 3 (1.0) | 0.148 (0.024-0.921) | **0.041** | 0.052 | 2 (0.8) | 0.318 (0.054-1.863) | 0.204 | 0.272 |
| TT/AA | 3 (1.0) | 1 (0.3) | N/A |  |  | 0 (0.0) | N/A |  |  | 1 (0.4) | N/A |  |  |
| TT/AG | 2 (0.7) | 1 (0.3) | N/A |  |  | 1 (0.3) | N/A |  |  | 0 (0.0) | N/A |  |  |
| TT/GG | 15 (5.0) | 16 (4.8) | 0.448 (0.118-1.705) | 0.239 | 0.319 | 12 (4.0) | 0.570 (0.143-2.278) | 0.427 | 0.319 | 3 (1.3) | 0.154 (0.015-1.586) | 0.116 | 0.186 |
| *MUC4* rs882605 G>T/rs2688513 A>G |  |  |  |  |  |  |  |  |  |  |  |  |  |
| GG/AA | 220 (73.3) | 262 (78.0) | 1.000(reference) |  |  | 154 (51.3) | 1.000(reference) |  |  | 104 (43.7) | 1.000(reference) |  |  |
| GG/AG | 27 (9.0) | 20 (6.0) | 0.309 (0.117-0.818) | **0.018** | **0.048** | 4 (1.3) | 0.092 (0.021-0.403) | **0.002** | **0.005** | 14 (5.9) | 0.665 (0.232-1.91) | 0.449 | 0.711 |
| GG/GG | 0 (0.0) | 1 (0.3) | N/A |  |  | 0 (0.0) | N/A |  |  | 1 (0.4) | N/A |  |  |
| GT/AA | 28 (9.3) | 18 (5.4) | 0.749 (0.199-2.829) | 0.670 | 0.915 | 9 (3.0) | 0.523 (0.098-2.789) | 0.448 | 0.597 | 8 (3.4) | 0.968 (0.218-4.310) | 0.966 | 0.966 |
| GT/AG | 121 (40.3) | 142 (42.3) | 0.964 (0.573-1.624) | 0.891 | 0.915 | 79 (26.3) | 0.741 (0.410-1.342) | 0.323 | 0.517 | 59 (24.8) | 1.158 (0.619-2.168) | 0.646 | 0.738 |
| GT/GG | 4 (1.3) | 3 (0.9) | 1.135 (0.110-1.701) | 0.915 | 0.915 | 1 (0.3) | 0.683 (0.039-1.875) | 0.793 | 0.793 | 2 (0.8) | 2.211 (0.183-6.769) | 0.533 | 0.711 |
| TT/AA | 1 (0.3) | 1 (0.3) | N/A |  |  | 0 (0.0) | N/A |  |  |  | N/A |  |  |
| TT/AG | 6 (2.0) | 2 (0.6) | 0.053 (0.003-1.052) | 0.054 | 0.108 | 2 (0.7) | 0.079 (0.004-1.570) | 0.096 | 0.192 | 0 (0.0) | N/A |  |  |
| TT/GG | 13 (4.3) | 15 (4.5) | 1.109 (0.203-6.068) | 0.905 | 0.915 | 11 (3.7) | 1.399 (0.240-8.139) | 0.709 | 0.793 | 3 (1.3) | 0.302 (0.020-4.542) | 0.386 | 0.711 |
| *MUC4* rs882605 G>T/rs2246901 A>C |  |  |  |  |  |  |  |  |  |  |  |  |  |
| GG/AA | 217 (72.3) | 255 (75.9) | 1.000(reference) |  |  | 147 (49.0) | 1.000(reference) |  |  | 104 (43.7) | 1.000(reference) |  |  |
| GG/AC | 28 (9.3) | 23 (6.8) | 0.597 (0.244-1.460) | 0.258 | 0.699 | 7 (2.3) | 0.258 (0.078-0.852) | **0.026** | 0.104 | 14 (5.9) | 0.862 (0.305-2.439) | 0.780 | 0.780 |
| GG/CC | 2 (0.7) | 5 (1.5) | N/A | 0.994 | 0.994 | 4 (1.3) | N/A | 0.994 | 0.994 | 1 (0.4) | N/A |  |  |
| GT/AA | 37 (12.3) | 17 (5.1) | 0.674 (0.216-2.104) | 0.497 | 0.938 | 10 (3.3) | 0.479 (0.125-1.832) | 0.282 | 0.701 | 7 (2.9) | 0.749 (0.184-3.054) | 0.687 | 0.780 |
| GT/AC | 106 (35.3) | 142 (42.3) | 1.087 (0.637-1.857) | 0.759 | 0.938 | 75 (25.0) | 0.785 (0.426-1.447) | 0.438 | 0.701 | 62 (26.1) | 1.412 (0.750-2.656) | 0.285 | 0.380 |
| GT/CC | 10 (3.3) | 4 (1.2) | 0.801 (0.116-5.513) | 0.821 | 0.938 | 4 (1.3) | 1.366 (0.192-9.724) | 0.755 | 0.953 | 0 (0.0) | N/A |  |  |
| TT/AA | 1 (0.3) | 1 (0.3) | N/A |  |  | 0 (0.0) | N/A |  |  | 1 (0.4) | N/A |  |  |
| TT/AC | 3 (1.0) | 1 (0.3) | 0.123 (0.003-4.759) | 0.262 | 0.699 | 1 (0.3) | 0.189 (0.004-8.154) | 0.386 | 0.701 | 0 (0.0) | N/A |  |  |
| TT/CC | 16 (5.3) | 16 (4.8) | 0.704 (0.164-3.028) | 0.637 | 0.938 | 12 (4.0) | 0.851 (0.19-3.819) | 0.834 | 0.953 | 3 (1.3) | 0.226 (0.019-2.642) | 0.236 | 0.378 |
| *MUC4* rs1104760 A>G/rs2688513 A>G |  |  |  |  |  |  |  |  |  |  |  |  |  |
| AA/AA | 186 (62.0) | 250 (74.4) | 1.000(reference) |  |  | 147 (49.0) | 1.000(reference) |  |  | 101 (42.4) | 1.000(reference) |  |  |
| AA/AG | 15 (5.0) | 9 (2.7) | 0.314 (0.077-1.278) | 0.106 | 0.121 | 3 (1.0) | 0.111 (0.016-0.763) | **0.025** | **0.040** | 6 (2.5) | 0.577 (0.123-2.715) | 0.486 | 0.486 |
| AA/GG | 1 (0.3) | 1 (0.3) | N/A |  |  | 0 (0.0) | N/A |  |  | 1 (0.4) | N/A |  |  |
| AG/AA | 52 (17.3) | 28 (8.3) | 0.151 (0.059-0.389) | **0.0001** | **0.0003** | 15 (5.0) | 0.156 (0.050-0.487) | **0.001** | **0.003** | 10 (4.2) | 0.146 (0.040-0.527) | **0.003** | **0.008** |
| AG/AG | 120 (40.0) | 150 (44.6) | 0.644 (0.381-1.087) | 0.099 | 0.121 | 78 (26.0) | 0.478 (0.262-0.872) | 0.016 | 0.032 | 66 (27.7) | 0.797 (0.429-1.480) | 0.472 | 0.486 |
| AG/GG | 3 (1.0) | 2 (0.6) | N/A |  |  | 0 (0.0) | N/A |  |  | 2 (0.8) | N/A |  |  |
| GG/AA | 11 (3.7) | 3 (0.9) | 0.137 (0.022-0.841) | **0.032** | 0.051 | 1 (0.3) | 0.102 (0.01-1.085) | 0.058 | 0.066 | 2 (0.8) | 0.17 (0.017-1.689) | 0.130 | 0.189 |
| GG/AG | 19 (6.3) | 5 (1.5) | 0.114 (0.020-0.654) | **0.015** | **0.030** | 4 (1.3) | 0.118 (0.016-0.869) | 0.036 | 0.048 | 1 (0.4) | 0.158 (0.014-1.743) | 0.132 | 0.189 |
| GG/GG | 13 (4.3) | 16 (4.8) | 0.684 (0.162-2.895) | 0.606 | 0.606 | 12 (4.0) | 0.946 (0.214-4.184) | 0.941 | 0.941 | 3 (1.3) | 0.149 (0.012-1.891) | 0.142 | 0.189 |
| *MUC4* rs1104760 A>G/rs2246901 A>C |  |  |  |  |  |  |  |  |  |  |  |  |  |
| AA/AA | 180 (60.0) | 243 (72.3) | 1.000(reference) |  |  | 143 (47.7) | 1.000(reference) |  |  | 99 (41.6) | 1.000(reference) |  |  |
| AA/AC | 20 (6.7) | 13 (3.9) | 0.372 (0.123-1.124) | 0.080 | 0.149 | 4 (1.3) | 0.149 (0.033-0.675) | **0.014** | **0.037** | 8 (3.4) | 0.469 (0.129-1.709) | 0.251 | 0.287 |
| AA/CC | 2 (0.7) | 4 (1.2) | N/A |  |  | 3 (1.0) | N/A |  |  | 1 (0.4) | N/A |  |  |
| AG/AA | 65 (21.7) | 28 (8.3) | 0.113 (0.047-0.272) | **<0.0001** | **0.0004** | 13 (4.3) | 0.087 (0.028-0.266) | **<0.0001** | **0.0004** | 12 (5.0) | 0.134 (0.044-0.411) | **0.0004** | **0.001** |
| AG/AC | 101 (33.7) | 148 (44.0) | 0.764 (0.435-1.341) | 0.348 | 0.398 | 76 (25.3) | 0.538 (0.282-1.027) | 0.060 | 0.096 | 66 (27.7) | 1.001 (0.520-1.928) | 0.998 | 0.998 |
| AG/CC | 9 (3.0) | 4 (1.2) | 0.957 (0.077-1.829) | 0.973 | 0.973 | 4 (1.3) | 1.545 (0.119-0.039) | 0.739 | 0.739 | 0 (0.0) | N/A |  |  |
| GG/AA | 10 (3.3) | 2 (0.6) | 0.132 (0.013-1.399) | 0.093 | 0.149 | 1 (0.3) | 0.223 (0.022-2.307) | 0.208 | 0.277 | 1 (0.4) | N/A |  |  |
| GG/AC | 16 (5.3) | 5 (1.5) | 0.162 (0.032-0.808) | **0.026** | 0.069 | 3 (1.0) | 0.101 (0.013-0.790) | **0.029** | 0.058 | 2 (0.8) | 0.327 (0.050-2.145) | 0.244 | 0.287 |
| GG/CC | 17 (5.7) | 17 (5.1) | 0.471 (0.126-1.757) | 0.262 | 0.349 | 13 (4.3) | 0.632 (0.162-2.461) | 0.508 | 0.581 | 3 (1.3) | 0.109 (0.010-1.254) | 0.075 | 0.120 |
| *MUC4* rs2688513 A>G/rs2246901 A>C |  |  |  |  |  |  |  |  |  |  |  |  |  |
| AA/AA | 234 (78.0) | 264 (78.6) | 1.000(reference) |  |  | 153 (51.0) | 1.000(reference) |  |  | 108 (45.4) | 1.000(reference) |  |  |
| AA/AC | 13 (4.3) | 13 (3.9) | 0.802 (0.209-3.071) | 0.747 | 0.854 | 6 (2.0) | 0.462 (0.089-2.403) | 0.358 | 0.463 | 5 (2.1) | 0.75 (0.140-4.014) | 0.737 | 0.737 |
| AA/CC | 2 (0.7) | 4 (1.2) | N/A |  |  | 4 (1.3) | N/A |  |  | 0 (0.0) | N/A |  |  |
| AG/AA | 21 (7.0) | 9 (2.7) | 0.219 (0.059-0.815) | **0.024** | 0.064 | 4 (1.3) | 0.146 (0.030-0.713) | **0.017** | **0.034** | 4 (1.7) | 0.269 (0.052-1.390) | 0.117 | 0.234 |
| AG/AC | 123 (41.0) | 151 (44.9) | 0.911 (0.548-1.515) | 0.719 | 0.854 | 77 (25.7) | 0.632 (0.351-1.141) | 0.128 | 0.205 | 69 (29.0) | 1.193 (0.654-2.177) | 0.566 | 0.647 |
| AG/CC | 10 (3.3) | 4 (1.2) | 0.282 (0.045-1.774) | 0.177 | 0.354 | 4 (1.3) | 0.453 (0.070-2.922) | 0.405 | 0.463 | 0 (0.0) | N/A |  |  |
| GG/AA | 0 (0.0) | 0 (0.0) | N/A |  |  | 0 (0.0) | N/A |  |  | 0 (0.0) | N/A |  |  |
| GG/AC | 1 (0.3) | 2 (0.6) | 0.838 (0.072-9.724) | 0.888 | 0.888 | 0 (0.0) | N/A |  |  | 2 (0.8) | 2.183 (0.187-5.529) | 0.534 | 0.647 |
| GG/CC | 16 (5.3) | 17 (5.1) | 1.366 (0.266-7.027) | 0.709 | 0.854 | 12 (4.0) | 1.797 (0.334-9.674) | 0.495 | 0.495 | 4 (1.7) | 0.425 (0.043-4.201) | 0.464 | 0.647 |
| A OR, adjusted odds ratio; 95% CI, 95% confidence interval. A AOR is adjusted by age, sex, hypertension, diabetes mellitus, body mass index, high density lipoprotein cholesterol. | | | | | | | | | | | | | |
