# Supplementary material for: Genetic variants of MUC4 are associated with susceptibility to and mortality of colorectal cancer and exhibit synergistic effects with LDL-C levels
Source: PLoS One. 2023 Jun 29;18(6):e0287768. doi: 10.1371/journal.pone.0287768 (PMC10310026; doi:10.1371/journal.pone.0287768)
Supplement: S4 Table — (DOCX) [file pone.0287768.s006.docx]

| **S4 Table. *MUC4* genotype frequencies and patient 3-year mortality in overall, colon, and rectum cancer** | | | | | | | | | | | | |
| --- | --- | --- | --- | --- | --- | --- | --- | --- | --- | --- | --- | --- |
| Genotypes | Total CRC (n=464) | Death (n=71) | Adjusted HR (95% CI) | *P* | Colon (n=260) | Death (n=39) | Adjusted HR (95% CI) | *P* | Rectum (n=192) | Death (n=30) | Adjusted HR (95% CI) | *P* |
| *MUC4* rs882605 G>T |  |  |  |  |  |  |  |  |  |  |  |  |
| GG | 283 (61.0) | 44 (62.0) | 1.000(reference) |  | 158 (60.8) | 119 (66.7) | 1.000(reference) |  | 119 (62.0) | 18 (60.0) | 1.000(reference) |  |
| GT | 163 (35.1) | 24 (33.8) | 0.740 (0.416-1.317) | 0.308 | 89 (34.2) | 69 (28.2) | 0.553 (0.230-1.329) | 0.188 | 69 (35.9) | 12 (40.0) | 0.950 (0.349-2.589) | 0.921 |
| TT | 18 (3.9) | 3 (4.2) | 0.751 (0.163-3.463) | 0.715 | 13 (5.0) | 4 (5.1) | 0.874 (0.170-4.504) | 0.873 | 4 (2.1) | 0 (0.0) | N/A |  |
| Dominant | 446 (96.1) | 68 (95.8) | 0.752 (0.429-1.318) | 0.321 | 247 (95.0) | 188 (94.9) | 0.626 (0.276-1.422) | 0.266 | 188 (97.9) | 30 (100.0) | 0.930 (0.342-2.530) | 0.887 |
| Recessive | 181 (39.0) | 27 (38.0) | 1.025 (0.242-4.340) | 0.973 | 102 (39.2) | 73 (33.3) | 1.010 (0.217-4.704) | 0.990 | 73 (38.0) | 12 (40.0) | N/A |  |
| *MUC4* rs1104760 A>G |  |  |  |  |  |  |  |  |  |  |  |  |
| AA | 260 (56.0) | 37 (52.1) | 1.000(reference) |  | 150 (57.7) | 108 (56.4) | 1.000(reference) |  | 108 (56.3) | 15 (50.0) | 1.000(reference) |  |
| AG | 180 (38.8) | 28 (39.4) | 0.931 (0.525-1.651) | 0.808 | 93 (35.8) | 78 (30.8) | 0.836 (0.353-1.979) | 0.685 | 78 (40.6) | 15 (50.0) | 1.050 (0.421-2.621) | 0.917 |
| GG | 24 (5.2) | 6 (8.5) | 1.843 (0.688-4.942) | 0.226 | 17 (6.5) | 6 (12.8) | 2.847 (0.934-8.675) | 0.067 | 6 (3.1) | 0 (0.0) | N/A |  |
| Dominant | 440 (94.8) | 65 (91.5) | 1.030 (0.603-1.758) | 0.915 | 243 (93.5) | 186 (87.2) | 1.223 (0.585-2.556) | 0.594 | 186 (96.9) | 30 (100.0) | 0.902 (0.368-2.213) | 0.822 |
| Recessive | 204 (44.0) | 34 (47.9) | 2.123 (0.831-5.421) | 0.117 | 110 (42.3) | 84 (43.6) | 3.703 (1.280-0.712) | **0.016** | 84 (43.8) | 15 (50.0) | N/A |  |
| *MUC4* rs2688513 A>G |  |  |  |  |  |  |  |  |  |  |  |  |
| AA | 281 (60.6) | 40 (56.3) | 1.000(reference) |  | 163 (62.7) | 113 (64.1) | 1.000(reference) |  | 113 (58.9) | 15 (50.0) | 1.000(reference) |  |
| AG | 164 (35.3) | 25 (35.2) | 0.912 (0.510-1.631) | 0.758 | 85 (32.7) | 73 (28.2) | 0.845 (0.364-1.963) | 0.697 | 73 (38.0) | 13 (43.3) | 1.377 (0.511-3.713) | 0.530 |
| GG | 19 (4.1) | 6 (8.5) | 3.853 (1.422-10.441) | **0.008** | 12 (4.6) | 6 (7.7) | 4.099 (1.075-5.633) | **0.040** | 6 (3.1) | 2 (6.7) | 10.341 (1.396-6.586) | **0.023** |
| Dominant | 445 (95.9) | 65 (91.5) | 1.093 (0.638-1.873) | 0.747 | 248 (95.4) | 186 (92.3) | 1.084 (0.510-2.304) | 0.834 | 186 (96.9) | 28 (93.3) | 1.715 (0.676-4.350) | 0.259 |
| Recessive | 183 (39.4) | 31 (43.7) | 4.086 (1.569-10.637) | **0.004** | 97 (37.3) | 79 (35.9) | 3.578 (0.986-2.985) | 0.054 | 79 (41.1) | 15 (50.0) | 7.884 (1.488-1.772) | **0.016** |
| *MUC4* rs2246901 A>C |  |  |  |  |  |  |  |  |  |  |  |  |
| AA | 273 (58.8) | 39 (54.9) | 1.000(reference) |  | 157 (60.4) | 112 (59.0) | 1.000(reference) |  | 112 (58.3) | 16 (53.3) | 1.000(reference) |  |
| AC | 166 (35.8) | 33 (46.5) | 0.871 (0.490-1.548) | 0.639 | 83 (31.9) | 76 (30.8) | 0.929 (0.408-2.117) | 0.862 | 76 (39.6) | 13 (43.3) | 0.978 (0.386-2.478) | 0.963 |
| CC | 25 (5.4) | 8 (11.3) | 1.815 (0.670-4.916) | 0.243 | 20 (7.7) | 4 (10.3) | 1.383 (0.411-4.657) | 0.602 | 4 (2.1) | 1 (3.3) | 12.368 (0.483-6.961) | 0.131 |
| Dominant | 439 (94.6) | 72 (101.4) | 0.987 (0.576-1.690) | 0.962 | 240 (92.3) | 188 (89.7) | 1.106 (0.532-2.302) | 0.788 | 188 (97.9) | 29 (96.7) | 1.104 (0.445-2.742) | 0.831 |
| Recessive | 191 (41.2) | 41 (57.7) | 2.139 (0.827-5.535) | 0.119 | 103 (39.6) | 80 (41.0) | 1.790 (0.575-5.573) | 0.317 | 80 (41.7) | 14 (46.7) | 8.022 (0.822-8.311) | 0.075 |
| HR, hazard ratio  HR is adjusted for age, sex, hypertension, diabetes mellitus, tumor size, lymph node metastasis, chemotherapy, smoking, and alcohol based on Cox-regression analysis. | | | | | | | | | | | | |
