# Supplementary material for: Genetic variants of MUC4 are associated with susceptibility to and mortality of colorectal cancer and exhibit synergistic effects with LDL-C levels
Source: PLoS One. 2023 Jun 29;18(6):e0287768. doi: 10.1371/journal.pone.0287768 (PMC10310026; doi:10.1371/journal.pone.0287768)
Supplement: S5 Table — (DOCX) [file pone.0287768.s007.docx]

| **S5 Table. *MUC4* polymorphism genotype frequencies and patient 3-year relapse in overall, colon, and rectum cancer** | | | | | | | | | | | | |
| --- | --- | --- | --- | --- | --- | --- | --- | --- | --- | --- | --- | --- |
| Genotypes | Total CRC (n=464) | Relapse (n=76) | Adjusted HR (95% CI) | *P* | Colon (n=260) | Relapse (n=39) | Adjusted HR (95% CI) | *P* | Rectum (n=192) | Relapse (n=35) | Adjusted HR (95% CI) | *P* |
| *MUC4* rs882605 G>T |  |  |  |  |  |  |  |  |  |  |  |  |
| GG | 283 (61.0) | 46 (60.5) | 1.000(reference) |  | 158 (60.8) | 28 (71.8) | 1.000(reference) |  | 119 (62.0) | 17 (48.6) | 1.000(reference) |  |
| GT | 163 (35.1) | 28 (36.8) | 0.774 (0.449-1.334) | 0.358 | 89 (34.2) | 9 (23.1) | 0.525 (0.216-1.273) | 0.156 | 69 (35.9) | 18 (51.4) | 1.252 (0.521-3.010) | 0.617 |
| TT | 18 (3.9) | 2 (2.6) | 0.532 (0.114-2.493) | 0.426 | 13 (5.0) | 2 (5.1) | 0.574 (0.110-3.010) | 0.514 | 4 (2.1) | 0 (0.0) | N/A |  |
| Dominant | 446 (96.1) | 74 (97.4) | 0.759 (0.444-1.295) | 0.314 | 247 (95.0) | 37 (94.9) | 0.588 (0.259-1.338) | 0.208 | 188 (97.9) | 35 (100.0) | 1.200 (0.501-2.873) | 0.684 |
| Recessive | 181 (39.0) | 30 (39.5) | 0.653 (0.153-2.785) | 0.567 | 102 (39.2) | 11 (28.2) | 0.852 (0.179-4.055) | 0.841 | 73 (38.0) | 18 (51.4) | N/A |  |
| *MUC4* rs1104760 A>G |  |  |  |  |  |  |  |  |  |  |  |  |
| AA | 260 (56.0) | 39 (51.3) | 1.000(reference) |  | 150 (57.7) | 22 (56.4) | 1.000(reference) |  | 108 (56.3) | 17 (48.6) | 1.000(reference) |  |
| GA | 180 (38.8) | 32 (42.1) | 1.005 (0.583-1.733) | 0.985 | 93 (35.8) | 13 (33.3) | 1.307 (0.591-2.887) | 0.511 | 78 (40.6) | 17 (48.6) | 0.796 (0.332-1.905) | 0.610 |
| GG | 24 (5.2) | 5 (6.6) | 1.675 (0.613-4.574) | 0.317 | 17 (6.5) | 4 (10.3) | 2.032 (0.569-7.263) | 0.278 | 6 (3.1) | 1 (2.9) | 0.808 (0.092-7.087) | 0.848 |
| Dominant | 440 (94.8) | 71 (93.4) | 1.075 (0.641-1.800) | 0.786 | 243 (93.5) | 35 (89.7) | 1.536 (0.740-3.186) | 0.252 | 186 (96.9) | 34 (97.1) | 0.751 (0.325-1.732) | 0.504 |
| Recessive | 204 (44.0) | 37 (48.7) | 1.761 (0.689-4.502) | 0.240 | 110 (42.3) | 17 (43.6) | 2.368 (0.736-7.622) | 0.150 | 84 (43.8) | 18 (51.4) | 0.724 (0.095-5.490) | 0.756 |
| *MUC4* rs2688513 A>G |  |  |  |  |  |  |  |  |  |  |  |  |
| AA | 281 (60.6) | 45 (59.2) | 1.000(reference) |  | 163 (62.7) | 27 (69.2) | 1.000(reference) |  | 113 (58.9) | 17 (48.6) | 1.000(reference) |  |
| GA | 164 (35.3) | 26 (34.2) | 0.821 (0.469-1.437) | 0.493 | 85 (32.7) | 9 (23.1) | 0.735 (0.308-1.754) | 0.489 | 73 (38.0) | 16 (45.7) | 0.902 (0.366-2.224) | 0.824 |
| GG | 19 (4.1) | 5 (6.6) | 2.894 (1.078-7.768) | **0.036** | 12 (4.6) | 3 (7.7) | 3.076 (0.743-2.733) | 0.123 | 6 (3.1) | 2 (5.7) | 1.213 (0.209-7.031) | 0.830 |
| Dominant | 445 (95.9) | 71 (93.4) | 0.962 (0.571-1.620) | 0.884 | 248 (95.4) | 36 (92.3) | 0.976 (0.457-2.083) | 0.949 | 186 (96.9) | 33 (94.3) | 0.970 (0.420-2.241) | 0.944 |
| Recessive | 183 (39.4) | 31 (40.8) | 2.748 (1.059-7.130) | **0.039** | 97 (37.3) | 12 (30.8) | 2.925 (0.792-0.812) | 0.109 | 79 (41.1) | 18 (51.4) | 1.452 (0.288-7.323) | 0.653 |
| *MUC4* rs2246901 A>C |  |  |  |  |  |  |  |  |  |  |  |  |
| AA | 273 (58.8) | 40 (52.6) | 1.000(reference) |  | 157 (60.4) | 23 (59.0) | 1.000(reference) |  | 112 (58.3) | 16 (45.7) | 1.000(reference) |  |
| CA | 166 (35.8) | 33 (43.4) | 0.954 (0.550-1.654) | 0.867 | 83 (31.9) | 10 (25.6) | 1.001 (0.417-2.404) | 0.999 | 76 (39.6) | 18 (51.4) | 0.974 (0.414-2.290) | 0.951 |
| CC | 25 (5.4) | 8 (10.5) | 2.012 (0.839-4.828) | 0.119 | 20 (7.7) | 6 (15.4) | 1.658 (0.558-4.924) | 0.365 | 4 (2.1) | 1 (2.9) | 0.348 (0.029-4.159) | 0.407 |
| Dominant | 439 (94.6) | 73 (96.1) | 1.092 (0.654-1.824) | 0.737 | 240 (92.3) | 33 (84.6) | 1.279 (0.616-2.654) | 0.512 | 188 (97.9) | 34 (97.1) | 0.924 (0.402-2.122) | 0.852 |
| Recessive | 191 (41.2) | 41 (53.9) | 2.086 (0.925-4.702) | 0.078 | 103 (39.6) | 16 (41.0) | 2.193 (0.834-5.768) | 0.113 | 80 (41.7) | 19 (54.3) | 0.642 (0.070-5.862) | 0.696 |
| HR, hazard ratio  HR is adjusted for age, sex, hypertension, diabetes mellitus, tumor size, lymph node metastasis, chemotherapy, smoking, and alcohol based on Cox-regression analysis. | | | | | | | | | | | | |
