# Supplementary material for: Genetic variants of MUC4 are associated with susceptibility to and mortality of colorectal cancer and exhibit synergistic effects with LDL-C levels
Source: PLoS One. 2023 Jun 29;18(6):e0287768. doi: 10.1371/journal.pone.0287768 (PMC10310026; doi:10.1371/journal.pone.0287768)
Supplement: S6 Table — (DOCX) [file pone.0287768.s008.docx]

| **S6 Table. *MUC4* polymorphism genotype frequencies and patient 5-year mortality in overall, colon, and rectum cancer** | | | | | | | | | | | | |
| --- | --- | --- | --- | --- | --- | --- | --- | --- | --- | --- | --- | --- |
| Genotypes | Total CRC (n=464) | Death (n=89) | Adjusted HR (95% CI) | *P* | Colon (n=260) | Death (n=47) | Adjusted HR (95% CI) | *P* | Rectum (n=192) | Death (n=39) | Adjusted HR (95% CI) | *P* |
| *MUC4* rs882605 G>T |  |  |  |  |  |  |  |  |  |  |  |  |
| GG | 283 (61.0) | 56 (78.9) | 1.000(reference) |  | 158 (60.8) | 32 (82.1) | 1.000(reference) |  | 119 (62.0) | 23 (76.7) | 1.000(reference) |  |
| GT | 163 (35.1) | 30 (42.3) | 0.643 (0.376-1.099) | 0.108 | 89 (34.2) | 13 (33.3) | 0.462 (0.205-1.040) | 0.063 | 69 (35.9) | 16 (53.3) | 0.807 (0.315-2.067) | 0.657 |
| TT | 18 (3.9) | 3 (4.2) | 0.729 (0.167-3.186) | 0.676 | 13 (5.0) | 2 (5.1) | 0.912 (0.189-4.397) | 0.909 | 4 (2.1) | 0 (0.0) | N/A |  |
| Dominant | 446 (96.1) | 86 (121.1) | 0.649 (0.385-1.094) | 0.106 | 247 (95.0) | 45 (115.4) | 0.528 (0.247-1.132) | 0.103 | 188 (97.9) | 39 (130.0) | 0.781 (0.306-1.990) | 0.606 |
| Recessive | 181 (39.0) | 33 (46.5) | 0.884 (0.212-3.682) | 0.866 | 102 (39.2) | 15 (38.5) | 1.095 (0.247-4.845) | 0.906 | 73 (38.0) | 16 (53.3) | N/A |  |
| *MUC4* rs1104760 A>G |  |  |  |  |  |  |  |  |  |  |  |  |
| AA | 260 (56.0) | 47 (66.2) | 1.000(reference) |  | 150 (57.7) | 26 (66.7) | 1.000(reference) |  | 108 (56.3) | 21 (70.0) | 1.000(reference) |  |
| AG | 180 (38.8) | 36 (50.7) | 0.896 (0.529-1.519) | 0.685 | 93 (35.8) | 16 (41.0) | 0.987 (0.465-2.092) | 0.972 | 78 (40.6) | 18 (60.0) | 0.803 (0.338-1.904) | 0.620 |
| GG | 24 (5.2) | 6 (8.5) | 1.442 (0.550-3.784) | 0.459 | 17 (6.5) | 5 (12.8) | 2.778 (0.929-8.305) | 0.069 | 6 (3.1) | 0 (0.0) | N/A |  |
| Dominant | 440 (94.8) | 83 (116.9) | 0.947 (0.577-1.552) | 0.829 | 243 (93.5) | 42 (107.7) | 1.290 (0.657-2.532) | 0.461 | 186 (96.9) | 39 (130.0) | 0.666 (0.287-1.549) | 0.348 |
| Recessive | 204 (44.0) | 42 (59.2) | 1.629 (0.645-4.110) | 0.304 | 110 (42.3) | 21 (53.8) | 3.474 (1.244-9.706) | **0.018** | 84 (43.8) | 18 (60.0) | N/A |  |
| *MUC4* rs2688513 A>G |  |  |  |  |  |  |  |  |  |  |  |  |
| AA | 281 (60.6) | 53 (74.6) | 1.000(reference) |  | 163 (62.7) | 31 (79.5) | 1.000(reference) |  | 113 (58.9) | 21 (70.0) | 1.000(reference) |  |
| AG | 164 (35.3) | 30 (42.3) | 0.764 (0.445-1.310) | 0.330 | 85 (32.7) | 13 (33.3) | 0.708 (0.326-1.538) | 0.385 | 73 (38.0) | 16 (53.3) | 0.933 (0.374-2.332) | 0.883 |
| GG | 19 (4.1) | 6 (8.5) | 3.235 (1.227-8.528) | **0.018** | 12 (4.6) | 3 (7.7) | 3.531 (0.955-3.054) | 0.060 | 6 (3.1) | 2 (6.7) | 6.496 (1.097-8.466) | **0.040** |
| Dominant | 445 (95.9) | 83 (116.9) | 0.903 (0.547-1.490) | 0.691 | 248 (95.4) | 44 (112.8) | 0.907 (0.449-1.832) | 0.787 | 186 (96.9) | 37 (123.3) | 1.174 (0.497-2.774) | 0.716 |
| Recessive | 183 (39.4) | 36 (50.7) | 3.392 (1.326-8.674) | **0.011** | 97 (37.3) | 16 (41.0) | 3.392 (0.980-1.743) | 0.055 | 79 (41.1) | 18 (60.0) | 5.848 (1.175-9.111) | **0.032** |
| *MUC4* rs2246901 A>C |  |  |  |  |  |  |  |  |  |  |  |  |
| AA | 273 (58.8) | 50 (70.4) | 1.000(reference) |  | 157 (60.4) | 27 (69.2) | 1.000(reference) |  | 112 (58.3) | 22 (73.3) | 1.000(reference) |  |
| AC | 166 (35.8) | 33 (46.5) | 0.753 (0.440-1.291) | 0.305 | 83 (31.9) | 14 (35.9) | 0.833 (0.386-1.796) | 0.642 | 76 (39.6) | 16 (53.3) | 0.709 (0.295-1.703) | 0.444 |
| CC | 25 (5.4) | 8 (11.3) | 2.078 (0.866-4.987) | 0.103 | 20 (7.7) | 6 (15.4) | 1.823 (0.620-5.355) | 0.277 | 4 (2.1) | 1 (3.3) | 5.709 (0.483-7.423) | 0.169 |
| Dominant | 439 (94.6) | 83 (116.9) | 0.897 (0.547-1.470) | 0.667 | 240 (92.3) | 41 (105.1) | 1.108 (0.569-2.156) | 0.764 | 188 (97.9) | 38 (126.7) | 0.796 (0.338-1.874) | 0.603 |
| Recessive | 191 (41.2) | 41 (57.7) | 2.479 (1.090-5.638) | **0.031** | 103 (39.6) | 20 (51.3) | 2.477 (0.937-6.546) | 0.069 | 80 (41.7) | 17 (56.7) | 4.769 (0.545-1.744) | 0.160 |
| HR, hazard ratio  HR is adjusted for age, sex, hypertension, diabetes mellitus, tumor size, lymph node metastasis, chemotherapy, smoking, and alcohol based on Cox-regression analysis. | | | | | | | | | | | | |
