# Supplementary material for: Genetic variants of MUC4 are associated with susceptibility to and mortality of colorectal cancer and exhibit synergistic effects with LDL-C levels
Source: PLoS One. 2023 Jun 29;18(6):e0287768. doi: 10.1371/journal.pone.0287768 (PMC10310026; doi:10.1371/journal.pone.0287768)
Supplement: S7 Table — (DOCX) [file pone.0287768.s009.docx]

| **S7 Table. *MUC4* polymorphism genotype frequencies and patient 5-year relapse in overall, colon, and rectum cancer** | | | | | | | | | | | | |
| --- | --- | --- | --- | --- | --- | --- | --- | --- | --- | --- | --- | --- |
| Genotypes | Total CRC (n=464) | Relapse (n=76) | Adjusted HR (95% CI) | *P* | Colon (n=260) | Relapse (n=39) | Adjusted HR (95% CI) | *P* | Rectum (n=192) | Relapse (n=35) | Adjusted HR (95% CI) | *P* |
| *MUC4* rs882605 G>T |  |  |  |  |  |  |  |  |  |  |  |  |
| GG | 283 (61.0) | 46 (64.8) | 1.000(reference) |  | 158 (60.8) | 28 (71.8) | 1.000(reference) |  | 119 (62.0) | 17 (56.7) | 1.000(reference) |  |
| GT | 163 (35.1) | 28 (39.4) | 0.762 (0.441-1.315) | 0.331 | 89 (34.2) | 9 (23.1) | 0.515 (0.212-1.252) | 0.145 | 69 (35.9) | 18 (60.0) | 1.226 (0.511-2.943) | 0.651 |
| TT | 18 (3.9) | 2 (2.8) | 0.531 (0.113-2.487) | 0.424 | 13 (5.0) | 2 (5.1) | 0.573 (0.109-3.004) | 0.513 | 4 (2.1) | 0 (0.0) | N/A |  |
| Dominant | 446 (96.1) | 74 (104.2) | 0.748 (0.438-1.278) | 0.291 | 247 (95.0) | 37 (94.9) | 0.58 (0.255-1.32) | 0.196 | 188 (97.9) | 35 (116.7) | 1.175 (0.491-2.811) | 0.719 |
| Recessive | 181 (39.0) | 30 (42.3) | 0.659 (0.155-2.808) | 0.575 | 102 (39.2) | 11 (28.2) | 0.871 (0.183-4.145) | 0.863 | 73 (38.0) | 18 (60.0) | N/A |  |
| *MUC4* rs1104760 A>G |  |  |  |  |  |  |  |  |  |  |  |  |
| AA | 260 (56.0) | 39 (54.9) | 1.000(reference) |  | 150 (57.7) | 22 (56.4) | 1.000(reference) |  | 108 (56.3) | 17 (56.7) | 1.000(reference) |  |
| AG | 180 (38.8) | 32 (45.1) | 0.994 (0.576-1.714) | 0.982 | 93 (35.8) | 13 (33.3) | 1.311 (0.594-2.894) | 0.505 | 78 (40.6) | 17 (56.7) | 0.774 (0.322-1.857) | 0.567 |
| GG | 24 (5.2) | 5 (7.0) | 1.658 (0.607-4.525) | 0.327 | 17 (6.5) | 4 (10.3) | 2.028 (0.567-7.249) | 0.279 | 6 (3.1) | 1 (3.3) | 0.711 (0.08-6.281) | 0.760 |
| Dominant | 440 (94.8) | 71 (100.0) | 1.064 (0.635-1.784) | 0.814 | 243 (93.5) | 35 (89.7) | 1.542 (0.744-3.196) | 0.246 | 186 (96.9) | 34 (113.3) | 0.734 (0.317-1.7) | 0.473 |
| Recessive | 204 (44.0) | 37 (52.1) | 1.768 (0.692-4.519) | 0.236 | 110 (42.3) | 17 (43.6) | 2.415 (0.753-7.75) | 0.140 | 84 (43.8) | 18 (60.0) | 0.735 (0.097-5.587) | 0.767 |
| *MUC4* rs2688513 A>G |  |  |  |  |  |  |  |  |  |  |  |  |
| AA | 281 (60.6) | 45 (63.4) | 1.000(reference) |  | 163 (62.7) | 27 (69.2) | 1.000(reference) |  | 113 (58.9) | 17 (56.7) | 1.000(reference) |  |
| AG | 164 (35.3) | 26 (36.6) | 0.814 (0.465-1.426) | 0.474 | 85 (32.7) | 9 (23.1) | 0.738 (0.309-1.763) | 0.497 | 73 (38.0) | 16 (53.3) | 0.884 (0.359-2.178) | 0.789 |
| GG | 19 (4.1) | 5 (7.0) | 2.862 (1.068-7.671) | **0.038** | 12 (4.6) | 3 (7.7) | 3.067 (0.741-2.702) | 0.124 | 6 (3.1) | 2 (6.7) | 1.185 (0.208-6.766) | 0.849 |
| Dominant | 445 (95.9) | 71 (100.0) | 0.955 (0.567-1.607) | 0.862 | 248 (95.4) | 36 (92.3) | 0.982 (0.460-2.096) | 0.963 | 186 (96.9) | 33 (110.0) | 0.952 (0.412-2.197) | 0.908 |
| Recessive | 183 (39.4) | 31 (43.7) | 2.737 (1.055-7.100) | **0.040** | 97 (37.3) | 12 (30.8) | 2.971 (0.806-0.953) | 0.104 | 79 (41.1) | 18 (60.0) | 1.428 (0.284-7.17) | 0.667 |
| *MUC4* rs2246901 A>C |  |  |  |  |  |  |  |  |  |  |  |  |
| AA | 273 (58.8) | 40 (56.3) | 1.000(reference) |  | 157 (60.4) | 23 (59.0) | 1.000(reference) |  | 112 (58.3) | 16 (53.3) | 1.000(reference) |  |
| AC | 166 (35.8) | 33 (46.5) | 0.946 (0.546-1.641) | 0.845 | 83 (31.9) | 10 (25.6) | 1.010 (0.421-2.425) | 0.982 | 76 (39.6) | 18 (60.0) | 0.953 (0.405-2.241) | 0.912 |
| CC | 25 (5.4) | 8 (11.3) | 2.002 (0.834-4.803) | 0.122 | 20 (7.7) | 6 (15.4) | 1.653 (0.556-4.914) | 0.368 | 4 (2.1) | 1 (3.3) | 0.356 (0.03-4.237) | 0.416 |
| Dominant | 439 (94.6) | 73 (102.8) | 1.084 (0.649-1.811) | 0.758 | 240 (92.3) | 33 (84.6) | 1.283 (0.618-2.662) | 0.506 | 188 (97.9) | 34 (113.3) | 0.904 (0.393-2.077) | 0.813 |
| Recessive | 191 (41.2) | 41 (57.7) | 2.079 (0.922-4.684) | 0.079 | 103 (39.6) | 16 (41.0) | 2.204 (0.837-5.805) | 0.112 | 80 (41.7) | 19 (63.3) | 0.634 (0.07-5.763) | 0.687 |
| HR, hazard ratio  HR is adjusted for age, sex, hypertension, diabetes mellitus, tumor size, lymph node metastasis, chemotherapy, smoking, and alcohol based on Cox-regression analysis. | | | | | | | | | | | | |
