# Supplementary material for: Genetic variants of MUC4 are associated with susceptibility to and mortality of colorectal cancer and exhibit synergistic effects with LDL-C levels
Source: PLoS One. 2023 Jun 29;18(6):e0287768. doi: 10.1371/journal.pone.0287768 (PMC10310026; doi:10.1371/journal.pone.0287768)
Supplement: S8 Table — (DOCX) [file pone.0287768.s010.docx]

| Characteristic | Association | AOR (95% CI) | Statistical power (%) |
| --- | --- | --- | --- |
| *MUC4* rs1104760 A>G | Table 2 | 0.537 (0.334-0.863) | 49.7 |
| *MUC4* rs1104760 A>G | Table 2 | 0.297 (0.121-0.730) | 97.5 |
| *MUC4* rs1104760 A>G | Table 2 | 0.493 (0.313-0.775) | 73.1 |
| *MUC4* rs1104760 A>G | Table 2 | 0.382 (0.163-0.898) | 77 |
| AOR, adjusted odds ratio. | | | |

**S8 Table. Statistical power of genetic association of less than 0.05 *p*-value in Table 2**
